# Differentiating Fetal Alcohol Spectrum Disorders From Attention‐Deficit Hyperactivity Disorder and Reviewing Diagnostic Guidelines

**DOI:** 10.1111/nyas.70343

**Published:** 2026-08-18

**Authors:** Todd J. Capes, Silvio Aldrovandi, Reshmail Khan, Virginia Amanatidou, Loukia Tsaprouni, Eirini Mavritsaki

**Affiliations:** ^1^ Birmingham City University Birmingham UK; ^2^ London South Bank University London UK; ^3^ Herefordshire and Worcestershire Health and Care National Health Service Trust Worcestershire UK

**Keywords:** attention‐deficit/hyperactivity disorder (ADHD), executive function, fetal alcohol spectrum disorders (FASDs), meta‐analysis, neuropsychology

## Abstract

Individuals with prenatal alcohol exposure (PAE) and fetal alcohol spectrum disorders (FASDs) present a range of neurodevelopmental deficits (e.g., inattention, hyperactivity, and executive dysfunction) which have shown marked overlap with attention‐deficit/hyperactivity disorder (ADHD), making differential diagnosis challenging. While rates of comorbidity are high, evidence has suggested there are important distinctions between neurodevelopmental phenotypes. To understand these distinctions, we evaluated whether proposed neurobehavioral disorder associated with prenatal alcohol exposure (ND‐PAE) criteria in the appendix of the *Diagnostic and Statistical Manual for Mental Disorders* (Fifth Edition) can differentiate FASD from ADHD. We conducted systematic searches across three databases (Medline, PsycINFO, PubMed) to identify studies comparing behavioral and cognitive functioning between FASD, ADHD, and healthy controls (HCs). Outcomes indicated FASD individuals have greater magnitude neurodevelopmental deficits than ADHD; however, no differences were found regarding the pattern of deficit because of methodological limitations, such as low *I* ^2^. Moreover, outcomes supported adaptive and neurocognitive (executive functioning) criteria but did not support self‐regulation criteria. These findings collectively underscore a need for clinicians to consider the magnitude of deficits presented to facilitate accurate recognition of PAE. Further research characterizing ND‐PAE criteria and differences in the magnitude of deficits between disorders may ultimately support more accurate differential diagnosis.

## 1 Introduction

The catastrophic effects of prenatal alcohol exposure (PAE) on fetal development were initially recognized over half a century ago [1]. PAE can result in fetal alcohol spectrum disorders (FASDs) [2], a constellation of disorders characterized by neurodevelopmental deficits, which may present with or without facial dysmorphia and physical abnormalities [3, 4]. However, accurately diagnosing FASD and attention‐deficit/hyperactivity disorder (ADHD) has been challenging due to the lack of definitive biomarkers, diagnostic tests, or neurodevelopmental phenotypes [5, 6]; underreporting or lack of reliable data on maternal prenatal alcohol consumption [7]; inconsistent presence and stability of facial dysmorphia [8, 9]; and because attention deficits and hyperactivity are viewed as core symptoms of both disorders [10, 11]. Accordingly, ADHD is the most commonly diagnosed comorbid disorder in individuals with a PAE [12, 13, 14, 15]. For example, research shows the frequency of ADHD diagnoses is linearly associated with the severity of the PAE [16, 17], and hence, some have considered these disorders to be related rather than distinct [10, 18]. Despite a general agreement that PAE increases ADHD‐like symptoms [19], others argue these symptoms do not meet criteria for a clinical diagnosis of ADHD [20, 21]. Consequently, the exact nature of the overlap and distinction between FASD and ADHD remains unclear [15]. Therefore, this research evaluates the proposed neurobehavioral disorder associated with prenatal alcohol exposure (ND‐PAE) guidelines [22, 23] to determine whether or not these criteria function as a specific neurobehavioral phenotype of PAE and can differentiate FASD and ADHD individuals.

To differentiate both disorders, several studies have attempted to understand the pattern of executive deficits specific to FASD and ADHD, respectively [24, 25]. Executive function refers to a family of top‐down cognitive processes involved in goal‐driven behaviors [26]. It has been widely accepted that three core executive functions exist: cognitive flexibility, inhibitory control, and working memory [27]. These executive functions provide the supervisory cognitive processing required for typical human functioning [28], and as such, numerous brain regions and networks, particularly those located within the prefrontal cortex, are involved [26, 29, 30]. Deficits in executive functioning are considered to be a hallmark feature of both disorders [31, 32]; however, the pattern and severity of dysfunction varies. For example, research has shown FASD individuals present deficits in each of the three core executive functions [24]. In contrast, 89% of individuals with ADHD demonstrate a deficit in one executive domain, whereas only a third present with deficits in two or more domains [33]. Moreover, when compared, FASD individuals presented greater magnitude deficits in each of the core executive functions than those with ADHD [24, 25]. Taken collectively, this suggests that differences between disorder phenotypes possibly lie within the magnitude of presenting behavioral and cognitive deficits.

Limited experimental studies have compared well‐defined FASD and ADHD groups on behavioral and cognitive functions related to executive function [34]. Research demonstrates that compared to ADHD, FASD individuals perform poorer on academic achievement [35, 36], adaptive behavior [37], executive function [24, 25], language [38], and social cognition [39]. By contrast, those with ADHD show poorer performance on fine and gross motor abilities [40] and hyperactivity [41]. Other functions have shown a more varied effect. For example, both populations perform poorly on standardized measures of attention; however, the underlying mechanism differs [36]. Individuals with FASD struggled most on encoding and shifting attention, while individuals with ADHD struggled most with focused and sustained attention [36], though this pattern has been challenged by new evidence [42]. However, research is less clear in regard to the differences in self‐regulation abilities between FASD and ADHD individuals. Further research identifying the differences in neurobehavioral outcomes between disorders is needed to facilitate more accurate differential diagnosis.

FASD continues to pose a significant diagnostic complexity. While the prevalence of FASD and its associated economic and societal costs are high [2, 43, 44], diagnostic rates remain low, with only ∼1%–2% of all affected individuals ever being correctly diagnosed [45, 46]. The low rate of diagnosis can be attributed to two main problems. First, it has commonly been assumed a full multidisciplinary evaluation is required to diagnose FASD [45]. Based on the demand in the UK alone, this would mean an additional 39,116 multidisciplinary evaluations—equating to 39 full‐time FASD teams—are required to deal with just new cases each year [18], which is an unfeasible approach for even the wealthiest of countries [44]. Second, many individuals do not exhibit PAE‐associated facial dysmorphia [8, 9]. Most of the complexity in diagnosing FASD can be attributed to the heterogeneous effect PAE has on brain functioning [47]. In accordance, despite several efforts to define groups of PAE‐associated deficits [48, 49], no specific neurodevelopmental phenotype of FASD has yet been established [6]. The various diagnostic guidelines developed have, therefore, produced varying sensitivities and specificities, leading to diagnostic inconsistency [50, 51, 52]. Many FASD individuals, therefore, are at risk of being undiagnosed or misidentified [53], which can cause symptoms to progress while increasing the likelihood of developing secondary disabilities in adulthood [54, 55]. Further research characterizing neurodevelopmental differences between FASD and ADHD individuals is required to facilitate more accurate and timely diagnosis, which may ultimately help reduce the economic, social, personal, and familial impacts related to PAE.

While several narrative reviews have attempted to elucidate the specific differences in neurodevelopmental phenotype between FASD and ADHD [32, 56], few studies have quantitatively substantiated these differences [24, 25]. Hence, this research aims to evaluate whether the proposed ND‐PAE diagnostic guidelines can accurately differentiate individuals with FASD and ADHD. The proposed ND‐PAE criteria (i.e., neurocognitive functioning, self‐regulation, and adaptive functioning) were selected as they focus exclusively on the neurobehavioral deficit associated with PAE [23] and because research has identified the need to develop diagnostic tools that do not rely on facial dysmorphia [32, 57]. Moreover, while ND‐PAE guidelines take into consideration the mental health symptoms associated with PAE, authors noted future research should attempt to understand whether these criteria can differentiate PAE‐affected individuals from other mental health disorders (i.e., ADHD) [23]. By comparing these disorders on the behavioral and cognitive functions outlined in ND‐PAE, this will help determine whether ND‐PAE criteria function as a specific neurobehavioral phenotype of PAE and can accurately differentiate FASD individuals from those with ADHD, or if further refinements are required.

The applied rationale for this research relates to the scope of clinical benefits early and accurate diagnosis has for FASD and ADHD individuals. Accurately identifying PAE early is important because it allows individuals to be referred down the most appropriate treatment pathway [9, 58, 59]. For example, PAE‐affected individuals have shown a decreased responsiveness to medication prescribed for treating ADHD in comparison to individuals with ADHD without a PAE [60]. Through improving diagnostic identification of PAE, individuals will be able to access appropriate early interventions which may help to prevent symptom progression and secondary disabilities developing [55, 61].

This research aimed to evaluate whether the proposed ND‐PAE criteria can accurately differentiate FASD and ADHD individuals. We compared behavioral and cognitive functioning between individuals with FASD to those with ADHD and healthy controls (HCs).

## 2 Methods

### 2.1 Protocol and Preregistration

The systematic review and meta‐analysis was conducted in adherence to the PRISMA guidelines [62]. The systematic review and meta‐analysis protocol was preregistered on PROSPERO (CRD42024519526).

### 2.2 Eligibility Criteria

To ensure methodological rigor and replicability, we introduced several inclusion and exclusion criteria for all studies identified for full‐text screen.

**Inclusion criteria**:

1. *Cohort population*: Each study was required to contain child, adolescent, and/or adult participants. All participants must have received a validated diagnosis of FASD or ADHD. Confirmation of diagnosis must be supported by a validated diagnostic instrument for ADHD (i.e., Connors 3; 4‐Digit Diagnostic Code, or equivalent measures), and this must be clearly stated in the study methodology.
2. *Research design*: Only case−control studies were considered eligible for inclusion. Each of the included studies must have contained a well‐defined HC comparison group without reasonable suspicion of FASD or ADHD. However, other comorbid mental health disorders were included to enhance sample heterogeneity and outcome generalizability.
3. *Outcome measures*: Each study was required to include standardized and validated measures of relevant behavioral and/or cognitive function. These functions included: academic achievement, adaptive behavior, affect regulation, attention, executive function, hyperactivity/impulsivity, language, memory, motor ability, and/or social cognition.
4. *Language and location*: No restrictions were placed on the participants’ language or their country of origin. In addition, we did not exclude studies based on the language that the text was written in. Studies not written in English were translated (where applicable).
5. *Year of publication*: We decided not to exclude studies based on year of publication to ensure data completeness and comprehensiveness and avoid potential biases.
6. *Publication type*: Included studies were required to be published in a peer‐reviewed journal to ensure data consistency and reporting standards.

### 2.3 Exclusion Criteria

1. *Intervention/treatment studies*: We excluded studies that evaluated the effectiveness of interventions and/or treatments, as our aim was to identify behavioral and cognitive differences between FASD and ADHD in the absence of any intervention or treatment. Baseline data were not included because individuals could have received prior treatment.
2. *Articles on diagnostic guidelines*: Publications that critiqued, reviewed, or focused on the development of diagnostic guidelines were excluded to ensure original empirical data and to remove the potential for data duplication.
3. *Preclinical or in‐utero studies*: Animal studies or studies where the child has not yet been born (i.e., placental) were excluded. Only studies that investigated the prenatal effects of alcohol on postnatal human function were included.
4. *Postnatal alcohol exposures*: Studies which investigated the nonprenatal effect of alcohol on postnatal outcomes were excluded because the review focused on the impact of PAE on behavioral and cognitive developmental outcomes.
5. *Secondary and gray literature*: Conference abstracts, meeting papers, preprints, dissertations and theses, and pre‐existing reviews and meta‐analyses were excluded, as the purpose of our review was to extract empirical data from full‐length peer‐reviewed studies. We excluded these studies to allow for a better standard of reporting. Reference lists of reviews were harvested for additional eligible studies.
6. *Meta‐analysis*: We excluded studies that we could not obtain the necessary data for (i.e., effect size, pooled standard error, sample size). Data for these studies are presented in the Supplementary Materials.

### 2.4 Search Strategy

The systematic search was conducted over three databases: Medline, PsycINFO, and PubMed. We conducted a further search on Google Scholar. Despite recommendations to sample the initial 200–300 articles on Google Scholar [63], to ensure comprehensiveness, we sampled the initial 1000 articles for FASD and ADHD. This enabled potentially eligible studies not indexed by traditional databases to be identified and included. All other searches (Medline, PsycINFO, and PubMed) were not restricted by the 1000 article cap. Reference lists of included studies, and reviews and meta‐analyses identified by searches were harvested to identify eligible studies not revealed in database searches.

All searches were conducted on March 21st, 2024. The search string used was consistent across databases and included keywords: “FASD” *OR* “F*etal Alcohol Spectrum Disorders” *OR* “FAS” *OR* “F*etal Alcohol Syndrome” *OR* “pFAS” *OR* “Partial F*etal Alcohol Syndrome” *OR* “ARND” *OR* “Alcohol*Related Neurodevelopmental Disorder” *AND* “ADHD” *OR* “Attention*Deficit*Hyperactivity Disorder” *AND* “Academic Achievement” *OR* “Adaptive Behaviour” *OR* “Functional Impairment” *OR* “Affect Regulation” *OR* “Attention” *OR* “Cognition” *OR* “Executive Function*” *OR* “Hyperactiv*” *OR* “Impulsiv*” *OR* “Language” *OR* “Memory” *OR* “Motor Abilit*” *OR* “Social Cognition”.

### 2.5 Study Selection

**FIGURE 1:**
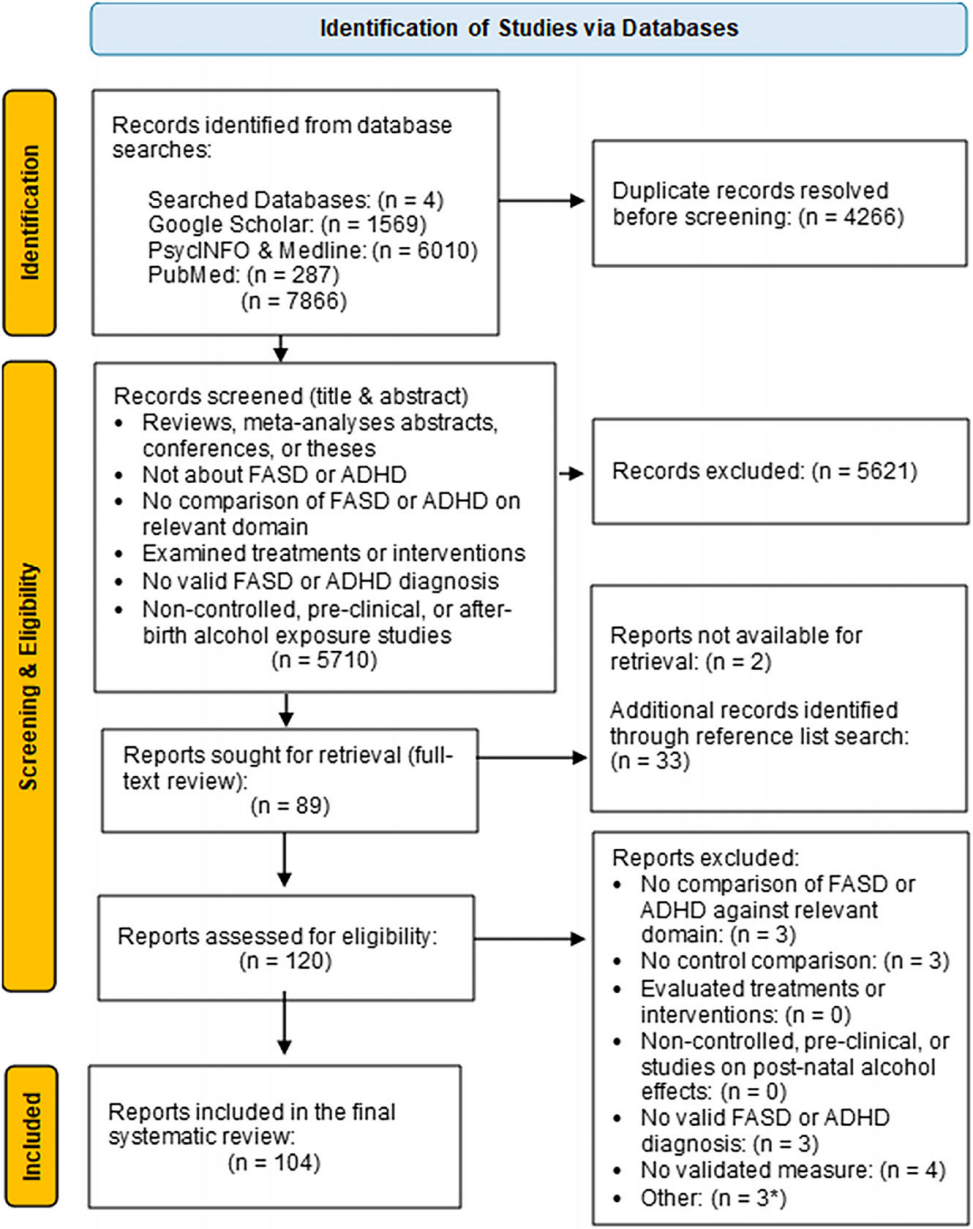
Flow chart of the Preferred Reporting Items for Systematic Reviews and Meta Analyses (PRISMA) [92]. This illustration details the search and screen procedure for the systematic review.

**FIGURE 2:**
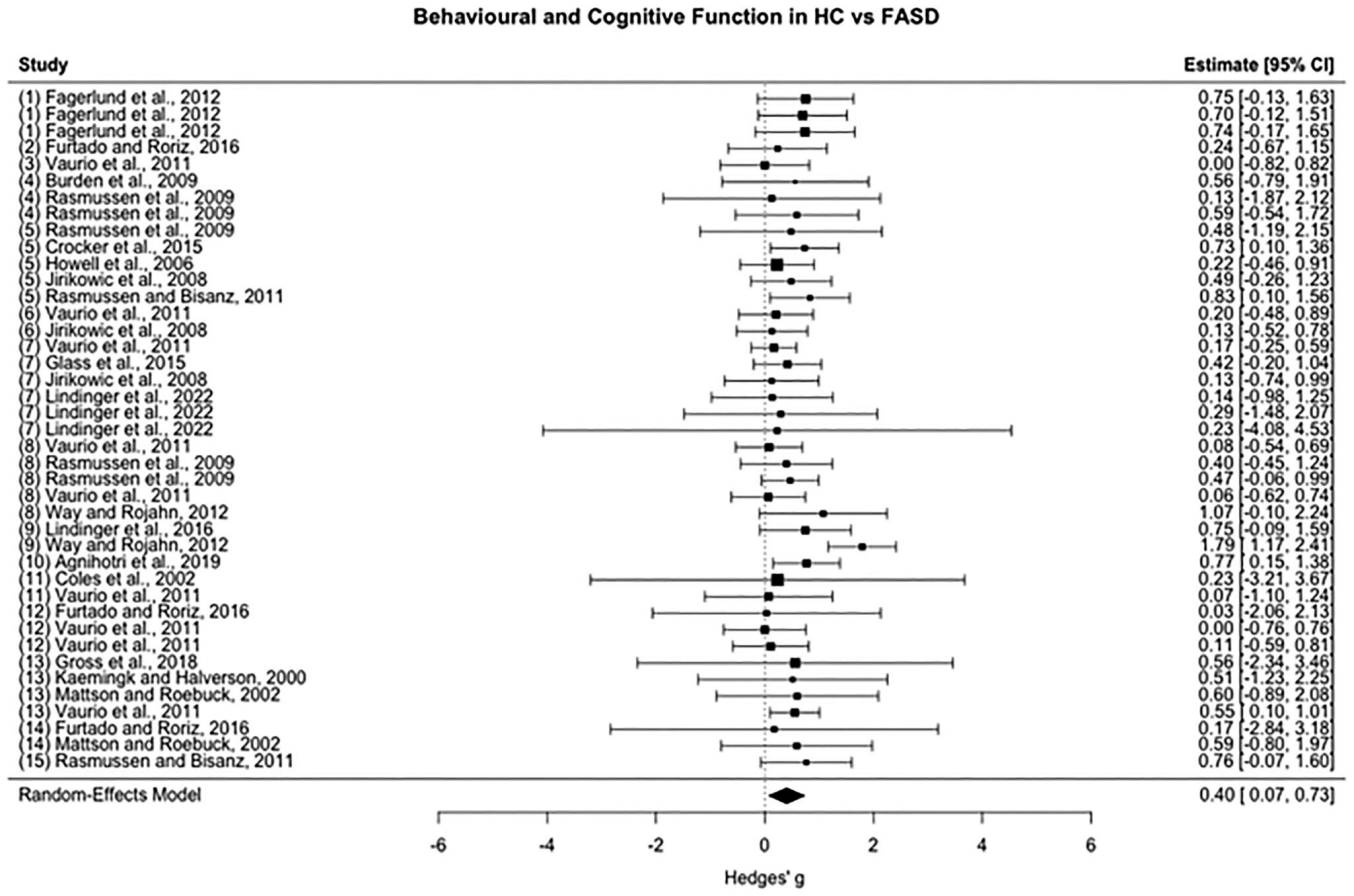
Meta‐analysis of behavioral and cognitive functions between fetal alcohol spectrum disorders (FASDs) and healthy controls (HCs). (1) Adaptive behavior, (2) cognitive flexibility, (3) expressive language, (4) inhibition, (5) mathematics, (6) motor function, (7) reading, (8) receptive language, (9) social cognition, (10) spatial working memory, (11) sustained attention, (12) verbal fluency, (13) verbal learning, (14) verbal memory, (15) working memory.

**FIGURE 3:**
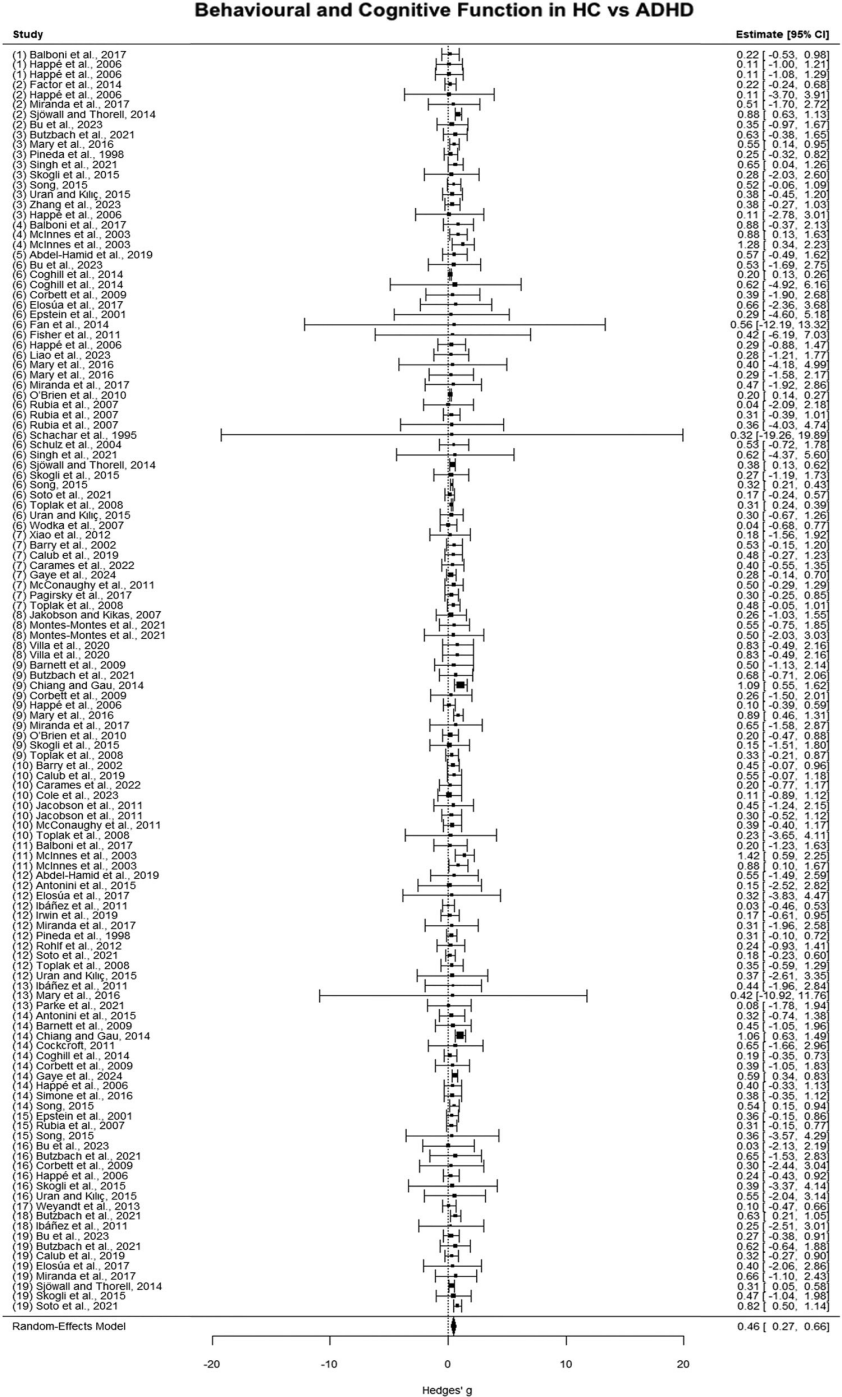
Meta‐analysis of behavioral and cognitive functions between attention‐deficit/hyperactivity disorder (ADHD) and healthy controls (HCs). (1) Adaptive behavior, (2) affect regulation, (3) cognitive flexibility, (4) design fluency, (5) expressive language, (6) inhibition, (7) mathematics, (8) motor function, (9) planning, (10) reading, (11) receptive language, (12) set‐shifting, (13) social cognition, (14) spatial working memory, (15) sustained attention, (16) verbal fluency, (17) verbal learning, (18) verbal memory, (19) working memory.

The study selection process is outlined in Figure 1, 2, 3, 4. Rayyan software was employed to screen articles. First, duplicate articles were removed. Next, the title and abstract were read independently by reviewers (T.J.C., E.M., S.A., R.K.). Each reviewer assessed study relevance in reference to inclusion/exclusion criteria. Studies were removed if the abstract failed to meet inclusion/exclusion criteria. All discrepancies in inclusion/exclusion were met with full team discussion.

**FIGURE 4:**
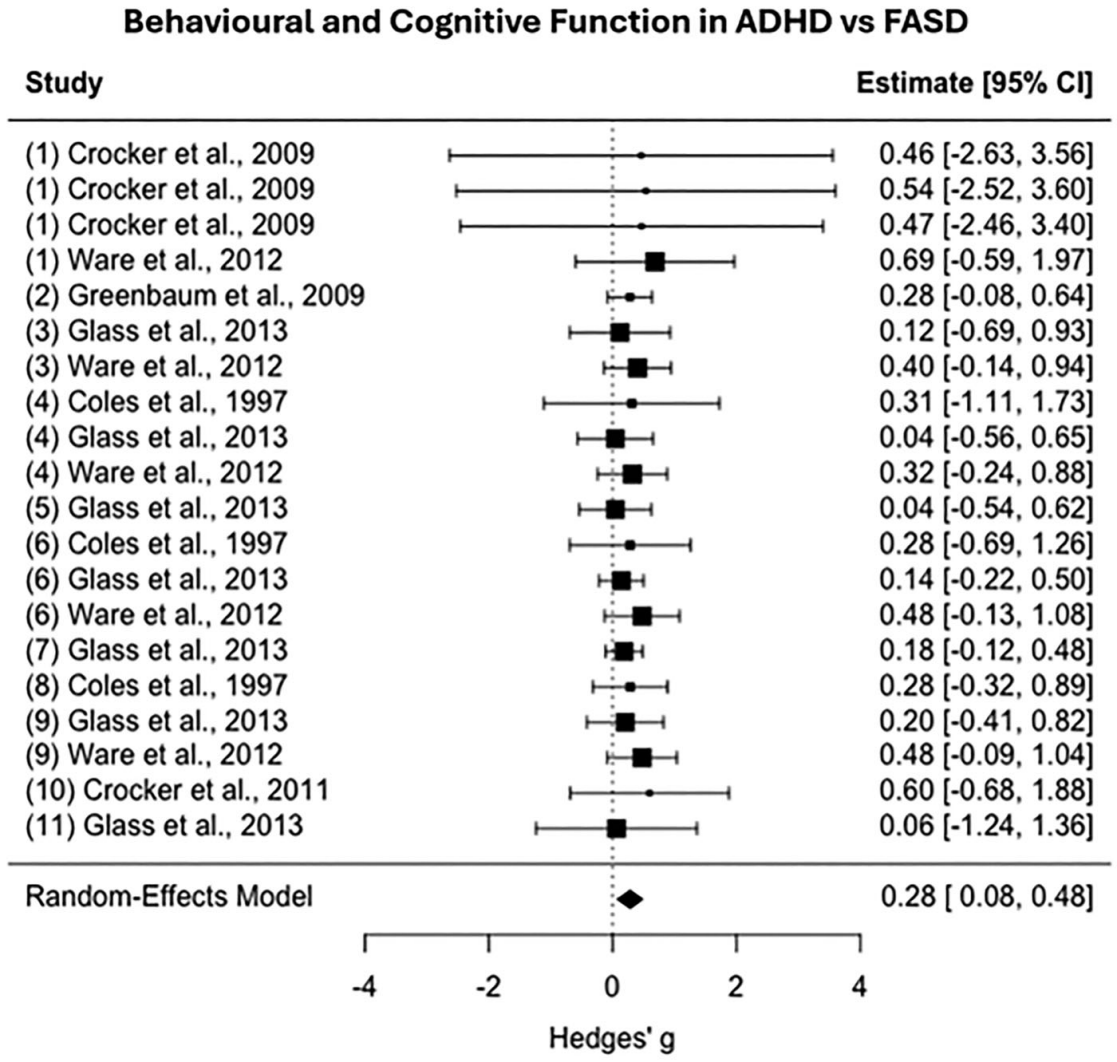
Meta‐analysis of behavioral and cognitive functions between fetal alcohol spectrum disorders (FASDs) and attention‐deficit/hyperactivity disorder (ADHD). (1) Adaptive behavior, (2) affect regulation, (3) design fluency, (4) inhibition, (5) planning, (6) set‐shifting, (7) spatial working memory, (8) sustained attention, (9) verbal fluency, (10) verbal learning, (11) working memory.

After identification and initial screening, full texts for the remaining 87 articles were read in full to ensure compliance with eligibility criteria. Full‐text screening was completed by the primary reviewer (T.J.C.). All studies that failed to meet inclusion criteria were excluded. From the applied screens, 71 studies were identified as suitable for inclusion. The search of reference lists identified a further 33 eligible studies. All included studies are summarized and listed in the reference list after Tables S1 and S2.

### 2.6 Data Collection Process

Of the 7866 (5710 after duplicate removal) articles identified, 104 studies remained eligible for the final systematic review. From the included 104 studies, we extracted data on sample demographics (i.e., age, gender, medication, comorbid disorders, lived environment, exposures, and socio‐economic status [SES]), study design (i.e., case−control, covariates), and key outcomes (i.e., behavioral and cognitive functions). Experimental measures implemented are summarized in Table 1.

**TABLE 1:** Experimental measure by neurobehavioral and neurocognitive subdomain.

Domain	Subdomain	Experimental measure(s)
Academic achievement	Mathematics	Kaufman‐Assessment Battery for Children [K‐ABC] Kaufmann Test of Educational Achievement [KTEA] Wechsler Individual Achievement Test [WIAT] Wide Range Achievement Test [WRAT] Woodcock−Johnson Task [WJ] Woodcock−McGrew−Werder Mini‐Battery of Achievement [WMWMB]
	Reading	Gray Oral Reading Tests [GORT] Kaufmann Test of Educational Achievement [KTEA] Test of Word Reading Efficiency [TWRE] Wechsler Individual Achievement Test [WIAT] Wide Range Achievement Test [WRAT] Woodcock Reading Mastery Test [WRMT] Woodcock−Johnson Task [WJ] Woodcock−McGrew−Werder Mini‐Battery of Achievement [WMWMB]
	Verbal learning	California Verbal Learning Test‐Children's [CVLT‐C] Wide Range Assessment of Memory and Learning [WRAML]
Adaptive behavior	**N/A**	Vineland Adaptive Behavior Scale [VABS]
Affect regulation	**N/A**	Behavior Rating Inventory of Executive Functioning‐Adults [BRIEF‐A—Affect Regulation Sub‐Test] Emotion Questionnaire [EQ] Minnesota Test of Affective Processing [MToAP] Positive and Negative Affect Scale‐Parent Report [P+NASPR] Strengths and Difficulties Questionnaire [SDQ] Trait Emotion Intelligence [TEI]
Attention	Overall	Attention Network Test [ANT]
	Sustained attention	Continuous Performance Task [CPT] Rapid Visual Information Processing Test [RVIPT—CANTAB] Sustained Attention to Response Task [SART] Sustained Attention Task [SAT—Amsterdam Neuropsychological Tasks Program] Test of Attentional Performance [TAP] Test of Variables of Attention [TOVA]
	Focused attention	Computerized Test of Attention [CTA] Focused Attention Task [FAT—Amsterdam Neuropsychological Tasks Program]
Executive function(s)	Cognitive flexibility	Trail Making Test‐B [TMT‐B—D‐KEFS] Wisconsin Card Sorting Task [WCST]
	Design fluency	Design Fluency Shifting [DFS] Design Fluency Task [DFT]
	Verbal fluency	Controlled Oral Word Association Test [COWAT] Letter + Category Fluency Test [L+CFT] Letter Fluency Task [LFT] Regensburger Word Fluency Test [RFT] Verbal Fluency Test [F‐A‐S] Verbal Fluency Test [VFT—D‐KEFS]
	Inhibition	Behavior Rating Inventory of Executive Functioning‐Adults [BRIEF‐A—Inhibition Sub‐Test] Day/Night Task [DNT] Go/no‐Go Task [GNG] Stroop Color‐Word Interference Task [Stroop—D‐KEFS] Cambridge Gambling Task [CGT—CANTAB] Five Digit Test [FDT] Stop Signal Task [StST] Stop Task [ST] Whispers Task [WT]
	Planning	Behavior Rating Inventory of Executive Functioning‐Adults [BRIEF‐A—Planning Sub‐Test] Stockings of Cambridge [SoC—CANTAB] Tower of London [ToL—CANTAB] Tower Test [TT—D‐KEFS]
	Set‐shifting	Berg Card Sorting Task [BCST] Behavior Rating Inventory of Executive Functioning‐Adults [BRIEF‐A—Set‐Shifting Sub‐Test] Trail Making Test‐B [TMT‐B—D‐KEFS) Global‐Local Set‐Shifting Task [GLSST] Wisconsin Card Sorting Task [WCST]
	Working memory	Behavior Rating Inventory of Executive Functioning‐Adults [BRIEF‐A—Working Memory Sub‐Test] Digit Span Task [DST—WISC] N‐Back Test [N‐Back] Wechsler Abbreviated Scale of Intelligence [WASI] Wechsler Intelligence Scale for Children [WISC] Working Memory Test Battery‐Children [WMTB]
	Verbal working memory	Automated Working Memory Assessment [AWMA]
	Spatial working memory	Automated Working Memory Assessment [AWMA] Children's Counting Span Task [CCST] Spatial Span Task [SST] Spatial Working Memory Task [SWMT—CANTAB] Wechsler Intelligence Scale for Children [WISC]
Hyperactivity/impulsivity	**N/A**	***No reported experimental measures***.
Language	Expressive language	Boston Naming Test [BNT] Clinical Evaluation of Language Fundamentals [CELF] Comprehensive Receptive and Expressive Vocabulary Test [CREVT] Expressive Vocabulary Test [EVT] Vineland Adaptive Behavior Scale [VABS]
	Receptive language	Clinical Evaluation of Language Fundamentals [CELF] Comprehensive Receptive and Expressive Vocabulary Test [CREVT] Peabody Picture Vocabulary Test [PPV] Vineland Adaptive Behavior Scale [VABS]
	Spatial memory	Everyday Memory Questionnaire [EMQ] Wide Range Assessment of Memory and Learning [WRAML]
Memory	Verbal memory	California Verbal Learning Test [CVLT‐C] Rey Auditory Verbal Learning Task [RAVLT] Verbal Learning and Memory Test [VLMT]
Motor ability	Fine motor ability	Bruininks−Oseretsky Test of Motor Proficiency [BOTMP] Clinical Observations of Motor and Postural Ability [COMPS] Developmental Coordination Disorders Questionnaire [DCDQ] Grooved Pegboard Task [GPT] Movement Assessment Battery for Children [M‐ABC] Tower Building [TB]
	Gross motor ability	Bruininks−Oseretsky Test of Motor Proficiency [BOTMP] Clinical Observations of Motor and Postural Ability [COMPS] Developmental Coordination Disorders Questionnaire [DCDQ] Movement Assessment Battery for Children [M‐ABC]
Social cognition	**N/A**	Reading the Mind in the Eyes Task [REM] Social Responsiveness Scale [SRS] Mentalistic Interpretation Task [MIT] Social Problems Fluency Task [SPFT] Social Problems Resolution Task [SPR]

### 2.7 Risk of Bias Quality Assessment

Studies were independently rated (T.J.C.) using the Newcastle−Ottawa Scale (NOS) [64]. This scale scores methodological quality of case−control studies from 0 to 9. Studies are awarded a star (*) if quality criteria requirements for selection, comparability, and exposure are met [64]. For selection and exposure criterion, independent studies are given one * if categorized “a” (i.e., full and/or blind diagnostic assessment); if categorized “b” or “c,” no * is awarded. For comparability, one * is awarded if the study controlled for age, but two * are awarded if age plus other factors were controlled (i.e., full‐scale IQ, medication, smoking, substance abuse, SES, living environment, and comorbid disorders). Higher scores on NOS indicate better methodological quality and a lower risk of bias.

### 2.8 Data Analysis

Heterogeneous tasks were aggregated based upon their main outcome. For example, both the continuous performance task and the sustained attention to response task measure sustained attention and were, therefore, both grouped under “attention.” The same method was applied to each outcome measure identified across all studies. This aggregation strategy was chosen as it allows for empirical evaluation of each domain of ND‐PAE, while also allowing for maximal statistical power in the event that systematic searches identify only a limited number of studies comparing clinical populations.

Effect sizes used in the meta‐analysis were calculated using the *metafor* package for R, and weighted‐sample random effects model meta‐analysis was employed [65]. Weighted‐sample random effects model meta‐analysis assumes effect sizes are drawn from various populations, allows random and true population variation, and considers the sample size of each study on the overall outcome. Because several studies contributed multiple effect sizes, a multilevel modeling approach was applied to deal with effect‐size dependency. We calculated *I* ^2^ to understand true variation across included studies compared to variation based on sampling error [66]. A smaller percentage value for *I* ^2^ indicates observed variation in the forest plot is due to sampling error, rather than true variation in effect sizes across studies [66]. In the instance that the *I* ^2^ value is above 50% moderator, analyses will be conducted [67].

Three meta‐analyses were conducted to compare the behavioral and cognitive function of FASD, ADHD, and HC. In the first analysis, we compared the difference in behavioral and cognitive deficit between FASD and HC (Figure 2). For the second analysis, we compared the difference in behavioral and cognitive deficit between ADHD and HC (Figure 3). Finally, we compared differences in behavioral and cognitive deficit between FASD and ADHD (Figure 4). Hedge's *g* was calculated to understand the differences in behavior and cognition.

To ensure publication bias did not influence outcomes, we included the NOS risk of bias scale and performed Egger's test for funnel plot asymmetry [68]. Publication bias was visually and statistically inspected.

## 3 Results

### 3.1 Study Characteristics and Results for Individual Studies

Of the 7866 identified studies, 104 were identified as eligible by the systematic search (see Tables S1 and S2); however, only 85 studies contained suitable data for the meta‐analysis. A summary of the study characteristics is available in Table S1, experimental measures in Table 1, and NOS in Table S2. For individual *p*‐values and effect sizes, refer to the Supplementary Materials.

### 3.2 Risk of Bias in Studies

NOS scores are detailed in Table S2. The authors of NOS stipulate no guidance over an acceptable threshold for inclusion. However, systematic reviews and meta‐analyses employ a threshold: 0–3 low quality + severe risk of bias; 4–6 moderate quality + some risk of bias; and 7–9 high quality + low risk of bias [69]. Most included studies scored within the moderate range, and few studies were classified in the low/high quality range.

All 104 identified articles provided a validated diagnosis of FASD and/or ADHD. One FASD and five ADHD studies implemented a random control sample. Fifty‐seven studies implemented community controls. The remaining 47 studies failed to specify the type of control sample. For comparability, half of the studies controlled for age. This number reduced to 41 for studies that controlled for age plus additional covariates. Lastly, for exposure, researchers were blind to the diagnostic status of individuals in six studies. In the remaining 98 studies, either researchers were not blind to diagnostic status, or the diagnostic process was not described. All 104 studies used the same measure/task and procedure for each experimental group. Finally, the rate of completion was identical between FASD, ADHD, and HC in 52 studies. The other 52 studies either noted a difference or did not describe completion rates.

### 3.3 Comparison of FASD and HC

To examine for publication bias, we visually inspected the funnel plot and performed Egger's test for funnel plot asymmetry [68]. From these, there was evidence of potential publication bias, *t*(39) = −2.51, *p* = 0.017. However, as indicated by the NOS, very few of our studies demonstrated a significant risk of bias. In addition, *I* ^2^ was not >50% (0.00%) and, therefore, indicated variation was due to sampling error, rather than true variation in effect sizes across included studies [66]. For this reason, moderator analyses were not conducted [67].

To examine where behavioral and cognitive differences lie between FASD and HC, we compared data across the 21 studies identified (41 effect sizes, *N* = 1825, *M* = 1.95 effect sizes per study). The overall effect size revealed a significant difference between behavioral and cognitive deficits across FASD and HC (*g* = 0.40, 95% CI [0.07, 0.73]). In addition, we performed a categorical summary analysis which revealed, compared to HC, those with FASD were associated with small‐to‐moderate deficit in receptive language (*g* = 0.40 95% CI [0.05, 0.76]), a moderate deficit in mathematics (*g* = 0.50, 95% CI [0.19, 0.80]), a moderate‐to‐large deficit in adaptive behavior (*g* = 0.72, 95% CI [0.22, 1.23]), spatial working memory (*g* = 0.77, 95% CI [0.15, 1.38]), and verbal learning (*g* = 0.55, 95% CI [0.14, 0.97]), and a large deficit in social cognition (*g* = 1.42, 95% CI [0.92, 1.92]). All other comparisons were nonsignificant.

### 3.4 Comparison of ADHD and HC

To examine any potential publication bias, we visually inspected the funnel plot and conducted Egger's test for funnel plot asymmetry [68]. There was evidence of potential publication bias, *t*(124) = 2.39, *p* = 0.018. However, as per NOS, very few of our studies indicated a severe risk of bias. *I* ^2^ was not >50% (8.82%), which indicated variation was due to sampling error, instead of true variation in the effect sizes of studies included [66]. For this reason, moderator analyses were not performed [67].

To examine where behavioral and cognitive differences lie between ADHD and HC, we compared data across the 58 studies identified (126 effect sizes, *N* = 5074, *M* = 2.17 effect sizes per study). The overall effect size revealed a significant difference between behavioral and cognitive deficits across ADHD and HC (*g* = 0.46, 95% CI [0.27, 0.66]). We also performed a categorical summary analysis that revealed, in comparison to HC, ADHD was associated with a small‐to‐moderate deficit in inhibition (*g* = 0.26, 95% CI [0.19, 0.33]), mathematics (*g* = 0.39, 95% CI [0.16, 0.63]), reading (*g* = 0.39, 95% CI [0.10, 0.69]), cognitive flexibility (*g* = 0.47, 95% CI [0.25, 0.70]), and working memory (*g* = 0.48, 95% CI [0.29, 0.67]), a moderate‐to large deficit in affect regulation (*g* = 0.71, 95% CI [0.47, 0.94]), planning (*g* = 0.56, 95% CI [0.33, 0.78]), spatial working memory (*g* = 0.58, 95% CI [0.40, 0.75]), and verbal memory (*g* = 0.62, 95% CI [0.19, 1.04]), and a large deficit in expressive language (*g* = 1.01, 95% CI [0.47, 1.54]), and in receptive language (*g* = 1.00, 95% CI [0.47, 1.54]). The remaining comparisons were nonsignificant.

### 3.5 Comparison of FASD and ADHD

To ensure results were not affected by publication bias, we visually inspected the funnel plot and conducted an Egger's test for funnel plot asymmetry [68]. No evidence indicated our results were subjected to significant influence from publication bias, *t*(18) = 1.68, *p* = 0.110. In addition, the NOS indicated very few studies indicated a risk of bias. *I* ^2^ was not >50% (0.00%), which indicated variation was due to sampling error, instead of true variation in the effect sizes of studies included [66]. For this reason, moderator analyses were not performed [67].

To examine where behavioral and cognitive differences lie between ADHD and HC, we compared data across the six studies identified (20 effect sizes, *N* = 1040, *M* = 3.33 effect sizes per study). The overall effect size revealed that the behavioral and cognitive impairments of FASD were significantly worse compared to ADHD (*g* = 0.28, 95% CI [0.08, 0.48]). In addition, we performed a categorical summary analysis; however, all comparisons were not significant.

## 4 Discussion

Our systematic review and meta‐analysis aimed to evaluate whether proposed ND‐PAE criteria can accurately differentiate FASD and ADHD. As reviewed, our outcomes showed that individuals with FASD experience greater magnitude neurodevelopmental deficits than ADHD. Compared to controls, FASD individuals demonstrated greater deficits in receptive language, mathematics, spatial working memory, verbal learning, and social cognition. In comparison to controls, individuals with ADHD showed greater deficits in inhibition, mathematics, reading, cognitive flexibility, working memory, affect regulation, planning, spatial working memory, verbal memory, expressive language, and receptive language. All other comparisons were nonsignificant. Therefore, although our outcomes were inconclusive regarding whether the pattern of deficit is uniform or not, differential diagnosis of FASD and ADHD may be more accurately achieved if clinicians consider the magnitude of presenting neurodevelopmental deficits.

Adaptive behavior concerns the ability to satisfy developmental standards in everyday living [70]. Our outcome is consistent with previous research, which found that FASD individuals showed more severe deficits on measures of adaptive behavioral functioning in comparison to those with ADHD [37, 71]. However, our data partially contrast Mattson's [32] model, who considered adaptive functioning deficit as a “shared impairment.” This discrepancy may be explained by disorder‐based differences in the developmental trajectory of adaptive functioning. Two studies revealed that adaptive functioning deficit for FASD individuals is a consequence of developmental arrest [72, 73]. By contrast, adaptive functioning for those with ADHD is developmentally delayed, but improves with age [37]. This perspective and our outcomes are consistent with a previous meta‐analysis [71], which revealed a larger effect size for adaptive functioning deficit in the FASD cohort in comparison to ADHD. However, it is well‐known that FASD and ADHD individuals are at an increased likelihood for adverse childhood experiences [74, 75], which have a powerful adverse impact on adaptive functioning outcomes, which may complicate diagnostic interpretations of the deficit presented. To enhance diagnostic precision of FASD and ADHD, it may be important to conduct longitudinal monitoring of adaptive functioning while assessing whether or not an improvement has occurred.

Difficulties with self‐regulation have been reported for both individuals with FASD and ADHD [76, 77]; however, our meta‐analysis only revealed significant deficits in self‐regulation for those with ADHD. This outcome could have occurred for two reasons. First, our systematic search yielded one study directly comparing FASD and ADHD samples [39]. The limited number of studies identified reduces statistical power to detect true effects, and outcomes regarding self‐regulation across disorders are inconclusive. Second, comparisons of self‐regulation in FASD are often confounded by biased sampling methods such as the use of clinical cohorts [78]. Clinical cohorts often have numerous other exposures beyond PAE, known to influence self‐regulation abilities [23, 79]. Without controlling for the additional confounding exposures, the strength of the effect between FASD and self‐regulation deficits rests liable to overestimation [78]. Although it is reasonable to hypothesize an association between PAE and impaired self‐regulation abilities, evidence is currently inconclusive whether this effect is due to PAE, or the additional adverse exposures faced by this population. Further research controlling for the additional confounding exposures of clinical populations is required before a causal effect can be established [80, 81].

While the majority of literature has focused on elucidating the neurocognitive differences between FASD and ADHD [24, 25, 32, 82], our outcomes did not support this in the traditional sense. Although our results revealed no differences regarding the pattern of deficit—likely due to low heterogeneity (*I* ^2^ values) and/or other methodological issues—we did find FASD individuals have higher magnitude neurocognitive deficits compared to ADHD individuals. This outcome, however, does not infer the existence of a uniform pattern of neurocognitive deficits across FASD and ADHD. Rather, these data imply, likewise to adaptive functioning criteria, that individuals with FASD may be better recognized if clinicians consider the magnitude of the neurocognitive deficit. For example, research has shown that at each SD threshold, executive function—a core neurocognitive domain—could predict PAE, although the remaining symptoms did not exceed chance [48], or have been found to be poor at predicting PAE [49]. Our outcomes, alongside the lack of endorsement reported by both validation studies, question the diagnostic efficacy of using the neurocognitive functioning domain in favor of other domains, such as adaptive functioning, to identify PAE‐affected individuals. Instead, it may be more appropriate to use neurocognitive deficits in executive function as a means to support the outcomes from other assessments (e.g., adaptive functioning) [83, 84], as collectively these exceed chance for predicting PAE.

As reviewed here, FASD and ADHD both cause adverse effects on neurodevelopmental functioning. Our outcomes showed that FASD individuals have greater magnitude deficits than ADHD [24]; however, no specific domain drove such effect. The higher magnitude deficit may be explained through mechanisms of alcohol teratogenesis. Ethanol interferes with a number of processes implicated in typical whole‐brain development, such as cell division, proliferation, growth, differentiation, and migration [85], which results in altered neural circuitry [86] and decreased neuronal plasticity [87]. For example, studies have shown PAE causes white matter abnormalities which reduce brain‐wide functional connectivity [86] above the effect of ADHD without PAE [88, 89]. The ability to distribute information processing to specialized brain centers is critical to ensure task demands can be executed. It is, therefore, not surprising that PAE‐affected individuals show weaker performance on measures assessing executive function—that depend on the integration of several brain systems [28]—than those with ADHD [24, 25]. The persistence of PAE‐related deficits, as opposed to delayed development observed for ADHD (i.e., adaptive functioning), may occur because alcohol exposure in‐utero is linked to long‐term reductions in frontal lobe plasticity [87]. Developmental plasticity of the frontal lobe is essential for adapting and regulating behavior, mental and physical health, as well as cognitive, social, and psychological development [26]. Our outcomes support the growing body of literature suggesting the difference between FASD and ADHD phenotype possibly lies within the magnitude of presenting deficits [24, 25, 37, 84, 90].

Our outcomes demonstrate adaptive behavior may offer the greatest significance for differentiating FASD and ADHD. Several validation studies of the proposed ND‐PAE criteria have uncovered that adaptive functioning yielded the largest area under curve value across 1.0, 1.5, and 2.0 SD thresholds [48, 49]. This demonstrates the adaptive functioning domain can differentiate individuals with and without a PAE and between the different levels of exposure. Our outcomes are consistent with both validation studies, in that we also found the magnitude of deficits may support more accurate identification of a PAE. Therefore, we support the inclusion of a threshold for deficits (i.e., the magnitude), as these may help to establish whether an individual has a PAE when a maternal history of prenatal alcohol consumption is unavailable while further clarifying the appropriate type and level of support required.

In contrast, results from our meta‐analysis were inconclusive regarding self‐regulation criteria despite that evidence of a deficit in this domain is required to satisfy a diagnosis of ND‐PAE [22, 23]. As mentioned, this likely occurred due to a limited number of studies and subsequent power to detect true effects, and/or sampling of clinical cohorts—which neither necessarily implies PAE has no effect on self‐regulation ability. Rather, what our outcomes indicate is that the inclusion of the self‐regulation domain as a diagnostic criterion required to be satisfied may be premature due to current methodological challenges restricting the ability to establish a causal effect [80, 81]. Two studies evaluating ND‐PAE criteria have reported evidence suggesting the self‐regulation domain can differentiate those with low PAE and nonexposed controls [48, 49]. Yet, in these studies, it is unclear whether impaired self‐regulation is caused by PAE due to the lack of control for ADHD comorbidity and/or additional exposures. Further research not confounded by the additional exposures faced by individuals with a PAE is needed to conclusively determine the contribution of the self‐regulation domain for a diagnosis of ND‐PAE. Until a causal effect of PAE on self‐regulation can be established, criteria for adaptive functioning may offer a more reliable way of identifying individuals with FASD and ADHD.

Although our meta‐analysis collapsed children, adolescents, and adults into one overall age category, we do recognize that each developmental stage presents varied symptomology [61], which may impact the sensitivity and/or specificity of proposed ND‐PAE criteria. For example, children with FASD and ADHD exhibit adaptive functioning deficit; however, individuals with ADHD show a higher rate of age‐related improvement [37, 71, 72, 73]. Relatedly, these data confer with a previous study, which found the likelihood of being diagnosed with ND‐PAE increased linear to developmental age [48]. Although this is important for ensuring more accurate recognition of a PAE, increased accuracy comes at the expense of worsening symptoms, emphasizing the need for timely diagnosis. Further studies evaluating ND‐PAE criteria by age categories are needed to understand whether criteria are adequately sensitive or require adjustment to accommodate for the age of each individual to ensure an accurate diagnostic outcome.

Outcomes of the systematic review and meta‐analysis should be interpreted in reference to the limitations contained. Few included studies directly compared behavioral and cognitive functioning between FASD and ADHD individuals. Second, most studies did not control for confounding variables known to influence the severity of deficits. Third, IQ was consistently controlled. Using IQ as a covariate or means for matching samples in studying behavior and/or cognition of neurodevelopmental populations has been shown to produce overcorrected, anomalous, and counterintuitive outcomes 91. By controlling IQ, this may have possibly reduced differences in the magnitude and/or pattern of deficit between FASD and ADHD, as well as decreasing the generalizability of our findings. Fourth, adulthood samples were underrepresented, making up 7.69% of the included studies. Fifth, we excluded secondary and/or gray literature, potentially restricting the scope of evidence. Sixth, *I* ^2^ values were low, which may have restricted power to detect true effects, such as differences in the pattern of deficit as opposed to clinical uniformity. Seventh, Egger's test for funnel plot asymmetry revealed a potential risk of bias, which possibly has implications for internal validity. Finally, ADHD studies rarely inquire about PAE; therefore, outcomes for the ADHD population may be confounded by PAE‐associated effects [23].

Despite the limitations, the study had several notable strengths. To our knowledge, this is the first systematic review and meta‐analysis to evaluate the proposed criteria for ND‐PAE. Second, our study undertook an expansive comparison of individuals with FASD and ADHD, sampling 7939 cases. Last, we included multiple measures to assess for risk of bias.

Future research should continue attempts to define the relationship between FASD and ADHD to improve diagnostic accuracy [15]. However, based on the limitations of our study, we recommend three important issues: (1) do not control for IQ (match/covariate) [91]; (2) control additional confounding variables (e.g., age, exposures, adverse experiences, medication, and SES); and (3) further study adult populations. Future research considering these factors will better identify the neurobehavioral phenotype of PAE and support more accurate differentiation of those with FASD and ADHD.

To date, no level of PAE is considered safe, as ethanol can readily permeate the placenta and negatively alter fetal development. However, accurately identifying those affected by PAE has remained challenging for several reasons, leading to underdiagnosis and misidentification of PAE‐affected individuals. As symptoms related to PAE show a marked similarity to ADHD, clinicians may mistakenly not recognize PAE. For this reason, we evaluated whether proposed ND‐PAE criteria can differentiate FASD and ADHD. Our outcomes showed that FASD individuals experience a greater magnitude of neurodevelopmental deficit than ADHD. However, we could not establish whether the pattern of deficit was uniform or varied due to several methodological limitations. Applied outcomes of our research indicate the need for clinicians to examine the magnitude of the neurodevelopmental deficit due to developmental brain differences when considering whether an individual should receive a diagnosis of ND‐PAE or ADHD. We hope our outcomes and the future directions offered will, in time, return improved diagnostic accuracy of FASD and eventually contribute to enhanced personal, familial, social, and economic outcomes.

## Author Contributions

All authors contributed toward the conception of the study. The design of the study was performed by Todd Capes, Eirini Mavritsaki, Silvio Aldrovandi, and Virginia Amanatidou. Review of the studies was performed by Todd Capes, Eirini Mavritsaki, Silvio Aldrovandi, and Reshmail Khan. Material analysis was performed by Todd Capes. The first draft of the manuscript was written by Todd Capes. Eirini Mavritsaki, Silvio Aldrovandi, and Virginia Amanatidou provided comments on the manuscript. All authors read and approved the final manuscript.

## Conflicts of Interest

The authors have no financial or nonfinancial interests to disclose.

## Supporting information




**Supplementary Materials**: nyas70343‐sup‐0001‐SuppMat.docx

## Data Availability

The data that support the findings of this study are available from the corresponding author, E.M., upon request.
