# Supplementary material for: Differentiating Fetal Alcohol Spectrum Disorders From Attention‐Deficit Hyperactivity Disorder and Reviewing Diagnostic Guidelines
Source: Ann N Y Acad Sci. 2026 Aug 18;1562(1):e70343. doi: 10.1111/nyas.70343 (PMC13488999; doi:10.1111/nyas.70343)
Supplement: Supplementary file 1 [file NYAS-1562-0-s001.docx]

| ***Table S1.*** Characteristics of included studies. | | | | | | |
| --- | --- | --- | --- | --- | --- | --- |
| Included Studies | FASD | | ADHD | | Control | |
| **Participants** |  |  |  |  |  |  |
| *N* | 1628 | | 4820 | | 4308 | |
| Age *M* | 9.01 | | 13.05 | | 12.47 | |
| Age Range | 4-18 | | 5-65 | | 2.5-65 | |
| **Gender** |  | |  | |  | |
| Male & Female | 71, 72, 73, 74, 75, 76, 77, 78, 79, 80, 81, 82, 83, 84, 85, 86, 87, 88, 89, 90, 91, 92, 93, 94, 96, 97, 98, 99, 100, 101, 102, 103, & 104 | | 1, 2, 3, 4, 5, 6, 7, 8, 9, 10, 11, 12, 13, 14, 16, 17, 18, 20, 21, 22, 23, 24, 25, 27, 28, 29, 31, 33, 34, 35, 36, 37, 38, 39, 42, 43, 44, 45, 47, 48, 49, 52, 53, 54, 55, 56, 57, 58, 59, 60, 61, 62, 63, 64, 65, 66, 67, 68, 70, 96, 97, 98, 99, 100, 101, 102, 103, & 104 | | 1, 2, 3, 4, 5, 6, 7, 8, 9, 10, 11, 12, 13, 14, 16, 17, 18, 20, 21, 22, 23, 24, 25, 27, 28, 29, 31, 33, 34, 35, 36, 37, 38, 39, 42, 43, 44, 45, 47, 48, 49, 52, 53, 54, 55, 56, 57, 58, 59, 60, 61, 62, 63, 64, 65, 66, 67, 68, 70, 71, 72, 73, 74, 75, 76, 77, 78, 79, 80, 81, 82, 83, 84, 85, 86, 87, 88, 89, 90, 91, 92, 93, 94, 96, 97, 98, 99, 100, 101, 102, 103, & 104 | |
| Male | 95 | | 15, 19, 30, 40, 41, 46, 50, 51, 69, & 95 | | 15, 19, 30, 40, 41, 46, 50, 51, 69, & 95 | |
| Female | **N/A** | | **N/A** | | **N/A** | |
| Not Specified | **N/A** | | 26, 32 | | 26, 32 | |
| **IQ** |  |  |  |  |  |  |
| *M* | ***73.24***  71, 72, 73, 74, 75, 76, 77, 80, 82, 83, 84, 85, 86, 87, 88, 91, 92, 94, 96, 97, 98, 99, 100, 101, 102, 103, & 104 | | ***99.11***  1, 2, 3, 5, 6, 7, 8, 9, 10, 12, 13, 14, 15, 16, 18, 21, 22, 25, 26, 28, 29, 30, 32, 35, 36, 37, 38, 39, 41, 43, 45, 46, 48, 49, 50, 51, 52, 53, 54, 55, 56, 58, 59, 60, 61, 62, 63, 67, 68, 69, 70, 96, 97, 98, 99, 100, 101, 102, 103, & 104 | | ***103.00***  1, 2, 3, 5, 6, 8, 9, 10, 12, 13, 14, 15, 16, 21, 22, 25, 26, 28, 29, 30, 32, 35, 36, 37, 38, 39, 41, 43, 45, 46, 48, 49, 50, 51, 52, 53, 54, 55, 56, 58, 59, 60, 61, 62, 63, 67, 68, 69, 70, 71, 72, 73, 74, 75, 76, 77, 80, 82, 83, 84, 85, 86, 87, 91, 92, 96, 97, 98, 99, 100, 101, 102, 103, & 104 | |
| Covaried/Matched in Analyses | 73, 74, 75, 76, 80, 81, 82, 86, 92, 96, 99, & 102 | | 1, 5, 10, 13, 17, 18, 20, 21, 25, 26, 27, 36, 37, 41, 45, 46, 51, 53, 54, 57, 58, 60, 61, 63, 67, 69, 96, 99, & 102 | | 1, 5, 10, 13, 17, 18, 20, 21, 25, 26, 27, 36, 37, 41, 45, 46, 51, 53, 54, 57, 58, 60, 61, 63, 67, 69, 73, 74, 75, 76, 80, 81, 82, 86, 92, 96, 99, & 102 | |
| Not Specified | 78, 79, 81, 89, 90, 93, & 95 | | 4, 11, 17, 19, 20, 23, 24, 27, 31, 33, 34, 40, 42, 44, 47, 57, 64, 65, 66, & 95 | | 4, 7, 11, 17, 18, 19, 20, 23, 24, 27, 31, 33, 34, 40, 42, 44, 47, 57, 64, 65, 66, 78, 79, 81, 88, 89, 90, 93, 94, & 95 | |
| **Medication** |  |  |  |  |  |  |
| Medication Naïve | **N/A** | | 1, 9, 11, 19, 22, 36, 47, 58, 64, 68 | | 11, 24, 25, 28, 37, 47, 49, 51, 58, 61, 62, 64, 67, 68, 93, & 99 | |
| Medication History | 78, 86, 93 | | 4, 5, 6, 10, 13, 14, 16, 17, 18, 20, 21, 23, 24, 25, 26, 28, 29, 20, 31, 32, 33, 34, 35, 37, 38, 39, 40, 41, 43, 45, 46, 48, 49, 50, 51, 52, 55, 56, 57, 59, 60, 61, 63, 65, 66, 67, 95, 98, 99, 100, 101, 102, & 103 | | 78, 86 | |
| Medication Covaried/Matched | 78 | | 17, 20, 24, 28, & 37 | | 17, 20, 24, 28, 37, & 78 | |
| Not Specified | 71, 72, 73, 74, 75, 76, 77, 79, 80, 81, 82, 83, 84, 85, 87, 88, 89, 90, 91, 92, 94, 95, 96, & 104 | | 2, 3, 8, 12, 15, 27, 42, 44, 53, 54, 62 69, 70, 96, & 104 | | 2, 3, 5, 6, 7, 8, 9, 10, 12, 13, 15, 18, 19, 21, 27, 30, 31, 32, 33, 34, 35, 36, 38, 39, 40, 41, 42, 43, 44, 45, 50, 53, 54, 55, 56, 57, 59, 60, 63, 65, 69, 70, 71, 72, 73, 74, 75, 76, 77, 79, 80, 81, 82, 83, 84, 85, 87, 88, 89, 90, 91, 92, 94, 95, 96, 97, 100, 101, 102, 103, & 104 | |
| **Comorbid Disorder(s)** |  |  |  |  |  | |
| Present (Disclosed) | 71, 78, 84, 85, 93, 94, 97, 98, 99, 100, 101, 102, 103, & 104 | | 6, 7, 10, 13, 15, 16, 18, 19, 20, 23, 24, 28, 29, 30, 32, 34, 37, 38, 39, 41, 43, 44, 45, 48, 49, 51, 52, 53, 57, 57, 60, 62, 63, 65, 66, 67, 99, & 102 | | 16, 20, 28, 29, 30, 32, 39, 41, 43, 52, 60, 78, 84, 85, 93 | |
| Present (Not Disclosed) | **N/A** | | 9 | | 23 | |
| No Comorbid Disorder(s) | **N/A** | | 1, 2, 4, 5, 14, 26, 31, 35, 36, 42, 47, 50, 54, 56, 59, 64, 69, 70, 100, & 101 | | 1, 2, 4, 5, 7, 10, 13, 14, 15, 18, 19, 26, 31, 34, 35, 36, 37, 42, 46, 47, 50, 53, 54, 56, 58, 59, 61, 62, 64, 69, 70, 71, 88, 99, 100, 101, & 102 | |
| Not Assessed | 72, 73, 74, 75, 76, 77, 79, 80, 81, 82, 83, 86, 87, 88, 89, 90, 91, 92, 95, & 96 | | 3, 8, 11, 12, 17, 21, 22, 25, 27, 33, 40, 46, 55, 61, 68, 95, 96, 97, 98, 103, & 104 | | 3, 6, 8, 9, 11, 12, 17, 21, 22, 24, 25, 27, 33, 38, 40, 44, 45, 48, 49, 51, 55, 57, 63, 65, 66, 67, 68, 72, 73, 74, 75, 76, 77, 79, 80, 81, 82, 83, 86, 87, 89, 90, 91, 92, 94, 95, 96, 97, 98, 103, & 104 | |
| **Lived Environment** |  |  |  |  |  |  |
| Biological Parents | 71, 74, 75, 80, 81, 86, 88, 89, 90, 91, 93, 94, & 99 | | 7, 24, & 99 | | 7, 24, 71, 74, 80, 81, 89, 90, 91, 94, & 99 | |
| Adoptive Parents | 71, 74, 75, 81, 86, 88, 89, 90, 91, 94, & 99 | | 7 | | 71, 74, 81, & 91 | |
| Foster Parents | 71, 74, 75, 81, 86, 88, 89, 90, 91, 94, & 99 | | **N/A** | | 71, 74, 81, & 91 | |
| Not-Specified | 72, 73, 76, 77, 78, 79, 82, 83, 84, 85, 87, 92, 95, 96, 97, 98, 100, 101, 102, 103, & 104 | | 1, 2, 3, 4, 5, 6, 8, 9, 10, 11, 12, 13, 14, 15, 16, 17, 18, 19, 20, 21, 22, 23, 25, 26, 27, 28, 29, 30, 31, 32, 33, 34, 35, 36, 37, 38, 39, 40, 41, 42, 43, 44, 45, 46, 47, 48, 49, 50, 51, 52, 53, 54, 55, 56, 57, 58, 59, 60, 61, 62, 63, 64, 65, 66, 67, 68, 69, 70, 95, 96, 97, 98, 100, 101, 102, 103, & 104 | | 1, 2, 3, 4, 5, 6, 8, 9, 10, 11, 12, 13, 14, 15, 16, 17, 18, 19, 20, 21, 22, 23, 25, 26, 27, 28, 29, 30, 31, 32, 33, 34, 35, 36, 37, 38, 39, 40, 41, 42, 43, 44, 45, 46, 47, 48, 49, 50, 51, 52, 53, 54, 55, 56, 57, 58, 59, 60, 61, 62, 63, 64, 65, 66, 67, 68, 69, 70, 72, 73, 75, 76, 77, 78, 79, 82, 83, 84, 85, 86, 87, 88, 89, 92, 93, 95, 96, 97, 98, 100, 101, 102, 103, & 104 | |
| **Exposure(s)** |  |  |  |  |  |  |
| Exposed (smoking/drugs) | 71, 72, 73, 80, 84, 85, 88, 94, 95, & 99 | | 1, 23, 29, 37, 48, & 99 | | 1, 23, 29, 72, 73, 80, 84, 85, 92, 95, & 99 | |
| Not Exposed (smoking/drugs) | **N/A** | | 19, 47, 49, 66, 68, 95, & 100 | | 19, 37, 47, 49, 66, 68, & 71 | |
| Not Specified | 74, 75, 76, 77, 78, 79, 81, 82, 83, 86, 87, 89, 90, 91, 92, 93, 96, 97, 98, 100, 101, 102, 103, & 104 | | 2, 3, 4, 5, 6, 7, 8, 9, 10, 11, 12, 13, 14, 15, 16, 17, 18, 20, 21, 22, 24, 25, 26, 27, 28, 30, 31, 32, 33, 34, 35, 36, 38, 39, 40, 41, 42, 43, 44, 45, 46, 50, 51, 52, 53, 54, 55, 56, 57, 58, 59, 60, 61, 62, 63, 64, 65, 67, 69, 70, 96, 97, 98, 101, 102, 103, & 104 | | 2, 3, 4, 5, 6, 7, 8, 9, 10, 11, 12, 13, 14, 15, 16, 17, 18, 20, 21, 22, 24, 25, 26, 27, 28, 30, 31, 32, 33, 34, 35, 36, 38, 39, 40, 41, 42, 43, 44, 45, 46, 48, 50, 51, 52, 53, 54, 55, 56, 57, 58, 59, 60, 61, 62, 63, 64, 65, 67, 69, 70, 74, 75, 76, 77, 78, 79, 81, 82, 83, 86, 87, 88, 89, 90, 91, 93, 94, 96, 97, 98, 100, 101, 102, 103, & 104 | |
| **Socio-Economic Status** |  |  |  |  |  |  |
| *M* | ***38.44***  71, 72, 74, 77, 84, 85, 86, 87, 91, 92, 96, 97, & 103 | | ***46.25***  15, 16, 19, 20, 28, 29, 32, 39, 43, 55, 60, 67, 96, 97, & 103 | | ***41.95***  15, 16, 19, 20, 28, 29, 32, 39, 43, 55, 60, 67, 71, 72, 74, 77, 84, 85, 86, 87, 91, 92, 96, 97, & 103 | |

*N/A* = not applicable.

This table identified the major demographic variables that influence the reliability of conclusions drawn. Here, the literature included in our review highlighted a major lack of (i.e., medication, lived environment teratogen exposure, and co-morbid disorder) and over (i.e., IQ) control of important variables that could help differentiate FASD and ADHD. The lack of control between disorders may exacerbate the expression of symptoms; over-control (i.e., IQ), however, could produce non-representative conclusions on neuropsychological function(s) (Dennis et al., 2009). Our review highlighted these issues, because interpretation may therefore become less biased and future research can ameliorate present issues.

***Table S2.*** Overview of scores on the Newcastle-Ottawa Risk of Bias scale (Wells et al., 2000) which assessed the methodological quality of case-control studies included within the final systematic review.

|  | **Study** | **Selection**  **(*max.* 4 stars)** | | | | **Comparability (*max.* 2 stars)** | **Exposure**  **(*max.* 3 stars)** | | |  |
| --- | --- | --- | --- | --- | --- | --- | --- | --- | --- | --- |
|  |  | **1** | **2** | **3** | **4** |  | **1** | **2** | **3** |  |
| ***ADHD+HC*** |  |  |  |  |  |  |  |  |  |  |
| 1 | Abdel‐Hamid et al. (2019) | * | *b* |  | * | ****** | *b* | * | *c* |  |
| 2 | Abo Elella et al. (2017) | * | *b* |  | * | ****** | *b* | * | * |  |
| 3 | Albrecht et al. (2013) | * | *b* |  | * | ***** | *c* | * | * |  |
| 4 | Amorim and Marques (2018) | * | *** |  | * |  | * | * | * |  |
| 5 | Antonini et al. (2015) | * | *b* |  | * | ****** | *b* | * | * |  |
| 6 | Arrington et al. (2022) | * | *b* | * | * |  | *c* | * | * |  |
| 7 | Balboni et al. (2017) | * | *b* |  | * | ****** | *b* | * | *c* |  |
| 8 | Baribeau et al. (2019) | * | *b* |  | * | ****** | *b* | * | * |  |
| 9 | Barnett et al. (2009) | * | *b* | * | * |  | *b* | * | * |  |
| 10 | Barry et al. (2002) | * | *b* | * | * |  | *b* | * | * |  |
| 11 | Bu et al. (2023) | * | *b* |  | * | ****** | *b* | * | * |  |
| 12 | Bullen et al. (2020) | * | *b* |  | * |  | *c* | * | *c* |  |
| 13 | Butzbach et al. (2021) | * | *b* | * | * | ****** | *b* | * | *c* |  |
| 14 | Çak et al. (2017) | * | *b* |  | * | ****** | *b* | * | *c* |  |
| 15 | Calub et al. (2019) | * | *b* | * | * |  | *b* | * | *c* |  |
| 16 | Carames et al. (2022) | * | *b* | * | * |  | *b* | * | * |  |
| 17 | Chiang and Gau (2014) | * | *b* |  | * | ****** | *b* | * | * |  |
| 18 | Cockcroft (2011) | * | *b* |  | * |  | *b* | * | * |  |
| 19 | Coghill et al. (2014) | * | *b* |  | * |  | *c* | * | * |  |
| 20 | Cole et al. (2023) | * | *b* | * | * | ****** | *b* | * | * |  |
| 21 | Corbett et al. (2009) | * | *b* |  | * |  | *b* | * | *c* |  |
| 22 | Elosúa et al. (2017) | * | *b* | * | * |  | *b* | * | * |  |
| 23 | Epstein et al. (2001) | * | *b* | * | * |  | *b* | * | * |  |
| 24 | Factor et al. (2014) | * | *b* | * | * | ****** | *c* | * | *c* |  |
| 25 | Fan et al. (2014) | * | *b* | * | * | ****** | *b* | * | * |  |
| 26 | Fisher et al. (2011) | * | *b* |  | * | ****** | *b* | * | *c* |  |
| 27 | Gaultney et al. (1999) | * | *b* |  | * | ****** | *b* | * | * |  |
| 28 | Gaye et al. (2024) | * | *b* | * | * |  | * | * | * |  |
| 29 | Gorman Bozorgpour et al. (2013) | * | *b* |  | * |  | *b* | * | *c* |  |
| 30 | Happé et al. (2006) | * | *b* | * | * |  | *b* | * | * |  |
| 31 | Ibáñez et al. (2011) | * | *b* |  | * | ****** | *b* | * | * |  |
| 32 | Irwin et al. (2019) | * | *b* | * | * |  | *b* | * | * |  |
| 33 | Jacobson et al. (2011) | * | *b* |  | * |  | *b* | * | * |  |
| 34 | Jakobson and Kikas (2007) | * | *b* | * |  | ****** | *b* | * | * |  |
| 35 | Johnstone et al. (2009) | * | *b* | * | * | ***** | *b* | * | *c* |  |
| 36 | Liao et al. (2023) | * | *b* | * | * | ****** | *b* | * | *c* |  |
| 37 | Marchetta et al. (2008a) | * | *b* | * | * |  | *b* | * | *c* |  |
| 38 | Mary et al. (2016) | * | *b* |  | * | ***** | *b* | * | * |  |
| 39 | McConaughy et al. (2011) | * | *b* |  | * |  | *b* | * | *c* |  |
| 40 | McInnes et al. (2003) | * | *b* | * | * |  | *b* | * | * |  |
| 41 | Miranda et al. (2017) | * | *b* |  | * | ****** | *b* | * | *c* |  |
| 42 | Montes-Montes et al. (2021) | * | *** | * | * | ****** | *b* | * | * |  |
| 43 | O’Brien et al. (2010) | * | *b* | * | * |  | *b* | * | * |  |
| 44 | Pagirsky et al. (2017) | * | *** |  | * |  | *b* | * | *c* |  |
| 45 | Parke et al. (2021) | * | *b* | * | * |  | *b* | * | *c* |  |
| 46 | Pineda et al. (1998) | * | *b* | * | * |  | *b* | * | *c* |  |
| 47 | Rajouyan et al. (2022) | * | *** |  | * | ***** | *b* | * | * |  |
| 48 | Rohlf et al. (2012) | * | *b* | * | * | ***** | *b* | * | * |  |
| 49 | Rubia et al. (2007) | * | *b* |  | * | ***** | *b* | * | *c* |  |
| 50 | Schachar et al. (1995) | * | *b* |  | * |  | *b* | * | * |  |
| 51 | Schulz et al. (2004) | * | *b* | * | * | ****** | *b* | * | *c* |  |
| 52 | Sciberras et al. (2014) | * | *** |  | * | ****** | *b* | * | *c* |  |
| 53 | Seymour et al. (2015) | * | *b* |  | * | ****** | *c* | * | * |  |
| 54 | Shallice et al. (2002) | * | *b* | * | * |  | *b* | * | *c* |  |
| 55 | Simone et al. (2016) | * | *b* |  | * |  | *b* | * | * |  |
| 56 | Singh et al. (2021) | * | *b* |  | * |  | *b* | * | * |  |
| 57 | Sjöwall and Thorell (2014) | * | *b* | * | * | ****** | *b* | * | *c* |  |
| 58 | Skogli et al. (2014) | * | *b* | * | * |  | *b* | * | *c* |  |
| 59 | Song (2015) | * | *b* |  | * | ****** | *b* | * | *c* |  |
| 60 | Soto et al. (2021) | * | *b* | * | * |  | *b* | * | * |  |
| 61 | Stavro et al. (2007) | * | *b* | * | * |  | *c* | * | *c* |  |
| 62 | Thoma et al. (2020) | * | *b* |  | * |  | *b* | * | *c* |  |
| 63 | Toplak et al. (2008) | * | *b* | * | * |  | *b* | * | *c* |  |
| 64 | Uran and Kılıç (2015) | * | *b* |  | * | ****** | *b* | * | * |  |
| 65 | Villa et al. (2020) | * | *b* | * | * | ***** | *b* | * | * |  |
| 66 | Weyandt et al. (2013) | * | *b* | * | * |  | *b* | * | *c* |  |
| 67 | Wodka et al. (2007) | * | *b* | * | * | ****** | *b* | * | *c* |  |
| 68 | Wu et al. (2023) | * | *b* | * | * | ****** | *b* | * | *c* |  |
| 69 | Xiao et al. (2012) | * | *b* | * | * | ****** | *b* | * | * |  |
| 70 | Zhang et al. (2023) | * | *b* | * | * | ****** | *b* | * | * |  |
| ***FASD+HC*** |  |  |  |  |  |  |  |  |  |  |
| 71 | Agnihotri et al. (2019) | * | *b* | * | * |  | *b* | * | * |  |
| 72 | Burden et al. (2009) | * | *b* | * | * |  | * | * | *c* |  |
| 73 | Coles et al. (2002) | * | *b* |  | * | ****** | *b* | *** | * |  |
| 74 | Crocker et al. (2015) | * | *b* | * | * | ****** | *b* | *** | *c* |  |
| 75 | Fagerlund et al. (2012) | * | *** |  | * |  | *b* | *** | *c* |  |
| 76 | Furtado and Roriz (2016) | * | *b* |  | * | ****** | * | *** | * |  |
| 77 | Glass et al. (2015) | * | *b* | * | * |  | *b* | *** | * |  |
| 78 | Green et al. (2009) | * | *b* |  | * | ****** | *b* | *** | *c* |  |
| 79 | Gross et al. (2018) | * | *b* |  | * |  | *b* | *** | * |  |
| 80 | Howell et al. (2006) | * | *b* |  | * |  | *b* | *** | * |  |
| 81 | Jirikowic et al. (2008) | * | *b* | * | * |  | *b* | *** | *c* |  |
| 82 | Kaemingk and Halverson (2000) | * | *b* |  | * | ***** | *b* | *** | *c* |  |
| 83 | Kerns et al. (2016) | * | *b* |  | * | ****** | *b* | *** | * |  |
| 84 | Lindinger et al. (2022) | * | *b* | * | * |  | *b* | *** | * |  |
| 85 | Lindinger et al. (2016) | * | *b* |  | * | ****** | *b* | *** | * |  |
| 86 | Mattson et al. (2006) | * | *b* | * | * | ***** | *b* | *** | *c* |  |
| 87 | Mattson and Roebuck (2002) | * | *b* | * | * |  | *b* | * | *c* |  |
| 88 | Rasmussen and Bisanz (2011) | * | *b* | * | * |  | *b* | * | *c* |  |
| 89 | Rasmussen et al. (2009a) | * | *b* | * | * | ***** | *b* | * | *c* |  |
| 90 | Rasmussen et al. (2009b) | * | *b* |  | * |  | *b* | * | *c* |  |
| 91 | Thomas et al. (1998) | * | *b* |  | * |  | *b* | * | *c* |  |
| 92 | Vaurio et al. (2011) | * | *b* | * | * | ****** | *b* | * | *c* |  |
| 93 | Way and Rojahn (2012) | * | *b* | * | * |  | *b* | * | * |  |
| 94 | Wyper and Rasmussen (2011) | * | *b* | * | * |  | *b* | * | *c* |  |
| ***FASD+ADHD+HC*** |  |  |  |  |  |  |  |  |  |  |
| 95 | Coles et al. (1997) | * | *b* |  | * |  | *b* | * | *c* |  |
| 96 | Crocker et al. (2009) | * | *b* | * | * | ****** | *b* | * | *c* |  |
| 97 | Crocker et al. (2011) | * | *b* | * | * | ****** | * | * | *c* |  |
| 98 | Glass et al. (2013) | * | *b* |  | * | ****** | *b* | * | * |  |
| 99 | Greenbaum et al. (2009) | * | *b* | * | * |  | *b* | * | * |  |
| 100 | Kooistra et al. (2009) | * | *b* | * | * |  | *b* | * | * |  |
| 101 | Kooistra et al. (2010) | * | *b* | * | * |  | *b* | * | *c* |  |
| 102 | Kooistra et al. (2011) | * | *b* | * | * | ****** | *b* | * | *c* |  |
| 103 | Vaurio et al. (2008) | * | *b* | * | * | ****** | * | * | * |  |
| 104 | Ware et al. (2012) | * | *b* |  | * |  | *b* | * | * |  |

*max.* = Maximum

Selection 1: *Participants with FASD or ADHD had a clinical diagnosis or diagnostic interview, assessment or psychometric evaluation to confirm diagnosis. Selection 2: * sampling of the control group was random; *b* sampling of the control group was non-random or otherwise not stated. Selection 3: *The control group consisted of a community sample. Selection 4: * Controls had no documented history of either FASD or ADHD (previous or current).

Comparability: *Matching/control of age in analysis, **matching/controlled for age + other covariates (inc. full-scale IQ, medication consumption, smoking, substance abuse, socio-economic status, environment, and co-morbid diagnosis).

Exposure 1: *Diagnostic assignment was completed blind to the diagnostic status of the participant; *b* diagnostic assignment was not completed blind to diagnostic status; *c* no description was provided on diagnostic assignment. Exposure 2: *The task administered to each population was identical; *b* different procedures were applied for the FASD /ADHD group and control group. Exposure 3: *Rate of task completion was the same for FASD /ADHD and control group; *c* rate of completion was different or not described.

This table provides an overview of the risk of bias for the included studies. The included studies were scored on three criteria (selection, comparability, and exposure). The majority of studies were considered moderate quality with some risk of bias; however, some of the research was considered high/low quality. The risk of bias scale was separated by neurodevelopmental disorders.

**References**

1.	Abdel‐Hamid, M., Niklewski, F., Heßmann, P., Guberina, N., Kownatka, M., Kraemer, M., Scherbaum, N., Dziobek, I., Bartels, C., Wiltfang, J., & Kis, B. (2019). Impaired Empathy but No Theory of Mind Deficits in Adult Attention Deficit Hyperactivity Disorder. *Brain and Behavior*, *9*(10), e01401. https://doi.org/10.1002/brb3.1401

2.	Abo Elella, E., Hassan, G. A. M., Sabry, W., Hendawy, H., Shorub, E., Zyada, F., & Medany, O. (2017). Trait emotional intelligence in a sample of Egyptian children with attention deficit hyperactivity disorder. *Child and Adolescent Mental Health*, *22*(4), 216–223. cmedm (32680413). https://doi.org/10.1111/camh.12236

3.	Albrecht, B., Brandeis, D., Uebel, H., Valko, L., Heinrich, H., Drechsler, R., Heise, A., Müller, U. C., Steinhausen, H.-C., Rothenberger, A., & Banaschewski, T. (2013). Familiality of neural preparation and response control in childhood attention deficit-hyperactivity disorder. *Psychological Medicine*, *43*(9), 1997–2011. cmedm (23200032). https://doi.org/10.1017/S003329171200270X

4.	Amorim, W. N., & Marques, S. C. (2018). Inhibitory control and cognitive flexibility in children with attention-deficit/hyperactivity disorder. *Psychology & Neuroscience*, *11*(4), 364–374. psyh (2018-60111-001). https://doi.org/10.1037/pne0000156

5.	Antonini, T. N., Becker, S. P., Tamm, L., & Epstein, J. N. (2015). Hot and Cool Executive Functions in Children with Attention-Deficit/Hyperactivity Disorder and Comorbid Oppositional Defiant Disorder. *Journal of the International Neuropsychological Society : JINS*, *21*(8), 584–595. cmedm (26416095). https://doi.org/10.1017/S1355617715000752

6.	Arrington, E. F., Alderson, R. M., Tarle, S. J., Roberts, D. K., Sarver, D. E., Sullivan, M. A., & Gette, J. A. (2022). Visuospatial working memory in attention-deficit/hyperactivity disorder: Characterizing path length and path crossings as mechanisms of impairment. *Neuropsychology*, *36*(5), 405–418. cmedm (35446052). https://doi.org/10.1037/neu0000812

7.	Balboni, G., Incognito, O., Belacchi, C., Bonichini, S., & Cubelli, R. (2017). Vineland-II adaptive behavior profile of children with attention-deficit/hyperactivity disorder or specific learning disorders. *Research in Developmental Disabilities*, *61*, 55–65. https://doi.org/10.1016/j.ridd.2016.12.003

8.	Baribeau, D. A., Dupuis, A., Paton, T. A., Hammill, C., Scherer, S. W., Schachar, R. J., Arnold, P. D., Szatmari, P., Nicolson, R., Georgiades, S., Crosbie, J., Brian, J., Iaboni, A., Kushki, A., Lerch, J. P., & Anagnostou, E. (2019). Structural neuroimaging correlates of social deficits are similar in autism spectrum disorder and attention-deficit/hyperactivity disorder: Analysis from the POND Network. *Translational Psychiatry*, *9*(1), 72. https://doi.org/10.1038/s41398-019-0382-0

9.	Barnett, R., Maruff, P., & Vance, A. (2009). Neurocognitive Function in Attention-Deficit–Hyperactivity Disorder with and Without Comorbid Disruptive Behaviour Disorders. *Australian & New Zealand Journal of Psychiatry*, *43*(8), 722–730. https://doi.org/10.1080/00048670903001927

10.	Barry, T. D., Lyman, R. D., & Klinger, L. G. (2002). Academic Underachievement and Attention-Deficit/ Hyperactivity Disorder: The Negative Impact of Symptom Severity on School Performance. *Journal of School Psychology*, *40*(3), 259–283. https://doi.org/https://doi.org/10.1016/S0022-4405(02)00100-0

11.	Bu, X., Gao, Y., Liang, K., Bao, W., Chen, Y., Guo, L., Gong, Q., Lu, H., Caffo, B., Mori, S., & Huang, X. (2023). Multivariate associations between behavioural dimensions and white matter across children and adolescents with and without attention‐deficit/hyperactivity disorder. *Journal of Child Psychology and Psychiatry*, *64*(2), 244–253. https://doi.org/10.1111/jcpp.13689

12.	Bullen, J. C., Swain Lerro, L., Zajic, M., McIntyre, N., & Mundy, P. (2020). A Developmental Study of Mathematics in Children with Autism Spectrum Disorder, Symptoms of Attention Deficit Hyperactivity Disorder, or Typical Development. *Journal of Autism and Developmental Disorders*, *50*(12), 4463–4476. cmedm (32306219). https://doi.org/10.1007/s10803-020-04500-9

13.	Butzbach, M., Fuermaier, A. B. M., Aschenbrenner, S., Weisbrod, M., Tucha, L., & Tucha, O. (2021). Metacognition in Adult Adhd: Subjective and Objective Perspectives on Self-Awareness of Cognitive Functioning. *Journal of Neural Transmission*, *128*(7), 939–955. https://doi.org/10.1007/s00702-020-02293-w

14.	Cak, H. T., Karaokur, R., Atasavun Uysal, S., Artik, A., Yildiz Kabak, V., Karakok, B., Sahan, N., Karaer, Y., Karabucak, B., Ozusta, S., & Cengel Kultur, E. (2017). Motor Proficiency in Children with Attention Deficit Hyperactivity Disorder: Associations with Cognitive Skills and Symptom Severity. *Turkish Journal of Psychiatry*. https://doi.org/10.5080/u22884

15.	Calub, C. A., Rapport, M. D., Friedman, L. M., & Eckrich, S. J. (2019). IQ and Academic Achievement in Children with ADHD: The Differential Effects of Specific Cognitive Functions. *Journal of Psychopathology and Behavioral Assessment*, *41*(4), 639–651. https://doi.org/10.1007/s10862-019-09728-z

16.	Carames, C. N., Irwin, L. N., & Kofler, M. J. (2022). Is there a relation between visual motor integration and academic achievement in school-aged children with and without ADHD? *Child Neuropsychology : A Journal on Normal and Abnormal Development in Childhood and Adolescence*, *28*(2), 224–243. cmedm (34494501). https://doi.org/10.1080/09297049.2021.1967913

17.	Chiang, H.-L., & Gau, S. S.-F. (2014). Impact of Executive Functions on School and Peer Functions in Youths with Adhd. *Research in Developmental Disabilities*, *35*(5), 963–972. https://doi.org/10.1016/j.ridd.2014.02.010

18.	Cockcroft, K. (2011). Working Memory Functioning in Children with Attention-Deficit/Hyperactivity Disorder (adhd): A Comparison Between Subtypes and Normal Controls. *Journal of Child & Adolescent Mental Health*, *23*(2), 107–118. https://doi.org/10.2989/17280583.2011.634545

19.	Coghill, D. R., Seth, S., & Matthews, K. (2014). A comprehensive assessment of memory, delay aversion, timing, inhibition, decision making and variability in attention deficit hyperactivity disorder: Advancing beyond the three-pathway models. *Psychological Medicine*, *44*(9), 1989–2001. https://doi.org/10.1017/S0033291713002547

20.	Cole, A. M., Chan, E. S. M., Gaye, F., Spiegel, J. A., Soto, E. F., & Kofler, M. J. (2023). Evaluating the Simple View of Reading for Children With Attention-Deficit/Hyperactivity Disorder. *Journal of Educational Psychology*, *115*(5), 700–714. cmedm (37873062). https://doi.org/10.1037/edu0000806

21.	Corbett, B. A., Constantine, L. J., Hendren, R., Rocke, D., & Ozonoff, S. (2009). Examining Executive Functioning in Children with Autism Spectrum Disorder, Attention Deficit Hyperactivity Disorder and Typical Development. *Psychiatry Research*, *166*(2–3), 210–222. https://doi.org/10.1016/j.psychres.2008.02.005

22.	Elosúa, M. R., Del Olmo, S., & Contreras, M. J. (2017). Differences in Executive Functioning in Children with Attention Deficit and Hyperactivity Disorder (ADHD). *Frontiers in Psychology*, *8*, 976. https://doi.org/10.3389/fpsyg.2017.00976

23.	Epstein, J. N., Johnson, D. E., Varia, I. M., & Conners, C. K. (2001). Neuropsychological Assessment of Response Inhibition in Adults With ADHD. *Journal of Clinical and Experimental Neuropsychology*, *23*(3), 362–371. https://doi.org/10.1076/jcen.23.3.362.1186

24.	Factor, P. I., Reyes, R. A., & Rosen, P. J. (2014). Emotional impulsivity in children with ADHD associated with comorbid—Not ADHD—symptomatology. *Journal of Psychopathology and Behavioral Assessment*, *36*(4), 530–541. psyh (2014-20449-001). https://doi.org/10.1007/s10862-014-9428-z

25.	Fan, L.-Y., Gau, S. S.-F., & Chou, T.-L. (2014). Neural correlates of inhibitory control and visual processing in youths with attention deficit hyperactivity disorder: A counting Stroop functional MRI study. *Psychological Medicine*, *44*(12), 2661–2671. cmedm (24451066). https://doi.org/10.1017/S0033291714000038

26.	Fisher, T., Aharon-Peretz, J., & Pratt, H. (2011). Dis-Regulation of Response Inhibition in Adult Attention Deficit Hyperactivity Disorder (adhd): An Erp Study. *Clinical Neurophysiology*, *122*(12), 2390–2399. https://doi.org/10.1016/j.clinph.2011.05.010

27.	Gaultney, J. F., Kipp, K., Weinstein, J., & McNeill, J. (1999). Inhibition and Mental Effort in Attention Deficit Hyperactivity Disorder. *Journal of Developmental and Physical Disabilities*, *2*, 105–114. https://doi.org/https://10.1023/A:1021890919601

28.	Gaye, F., Groves, N. B., Chan, E. S. M., Cole, A. M., Jaisle, E. M., Soto, E. F., & Kofler, M. J. (2024). Working memory and math skills in children with and without ADHD. *Neuropsychology*, *38*(1), 1–16. https://doi.org/10.1037/neu0000920

29.	Gorman Bozorgpour, E. B., Klorman, R., & Gift, T. E. (2013). Effects of subtype of attention-deficit/hyperactivity disorder in adults on lateralized readiness potentials during a go/no-go choice reaction time task. *Journal of Abnormal Psychology*, *122*(3), 868–878. cmedm (24016022). https://doi.org/10.1037/a0033992

30.	Happé, F., Booth, R., Charlton, R., & Hughes, C. (2006). Executive function deficits in autism spectrum disorders and attention-deficit/hyperactivity disorder: Examining profiles across domains and ages. In *Brain and Cognition* (Vol. 61, Issue 1, pp. 25–39). Elsevier BV. https://doi.org/10.1016/j.bandc.2006.03.004

31.	Ibáñez, A., Petroni, A., Urquina, H., Torrente, F., Torralva, T., Hurtado, E., Guex, R., Blenkmann, A., Beltrachini, L., Muravchik, C., Baez, S., Cetkovich, M., Sigman, M., Lischinsky, A., & Manes, F. (2011). Cortical deficits of emotional face processing in adults with ADHD: Its relation to social cognition and executive function. *Social Neuroscience*, *6*(5–6), 464–481. https://doi.org/10.1080/17470919.2011.620769

32.	Irwin, L. N., Kofler, M. J., Soto, E. F., & Groves, N. B. (2019). Do children with attention-deficit/hyperactivity disorder (ADHD) have set shifting deficits? *Neuropsychology*, *33*(4), 470–481. https://doi.org/10.1037/neu0000546

33.	Jacobson, L. A., Ryan, M., Martin, R. B., Ewen, J., Mostofsky, S. H., Denckla, M. B., & Mahone, E. M. (2011). Working memory influences processing speed and reading fluency in ADHD. *Child Neuropsychology*, *17*(3), 209–224. https://doi.org/10.1080/09297049.2010.532204

34.	Jakobson, A., & Kikas, E. (2007). Cognitive Functioning in Children With and Without Attention-Deficit/Hyperactivity Disorder With and Without Comorbid Learning Disabilities. *Journal of Learning Disabilities*, *40*(3), 194–202. https://doi.org/10.1177/00222194070400030101

35.	Johnstone, S. J., Barry, R. J., Markovska, V., Dimoska, A., & Clarke, A. R. (2009). Response inhibition and interference control in children with AD/HD: A visual ERP investigation. *International Journal of Psychophysiology*, *72*(2), 145–153. https://doi.org/10.1016/j.ijpsycho.2008.11.007

36.	Liao, W., Cao, L., Leng, L., Wang, S., He, X., Dong, Y., Yang, R., & Bai, G. (2023). Lack of Functional Brain Connectivity Was Associated with Poor Inhibition in Children with Attention-Deficit/Hyperactivity Disorder Using Near-Infrared Spectroscopy. *Frontiers in Psychiatry*, *14*, 1221242. https://doi.org/10.3389/fpsyt.2023.1221242

37.	Marchetta, N. D. J., Hurks, P. P. M., De Sonneville, L. M. J., Krabbendam, L., & Jolles, J. (2008). Sustained and Focused Attention Deficits in Adult ADHD. *Journal of Attention Disorders*, *11*(6), 664–676. https://doi.org/10.1177/1087054707305108

38.	Mary, A., Slama, H., Mousty, P., Massat, I., Capiau, T., Drabs, V., & Peigneux, P. (2016). Executive and attentional contributions to Theory of Mind deficit in attention deficit/hyperactivity disorder (ADHD). *Child Neuropsychology*, *22*(3), 345–365. https://doi.org/10.1080/09297049.2015.1012491

39.	McConaughy, S. H., Volpe, R. J., Antshel, K. M., Gordon, M., & Eiraldi, R. B. (2011). Academic and Social Impairments of Elementary School Children With Attention Deficit Hyperactivity Disorder. *School Psychology Review*, *40*(2), 200–225. https://doi.org/10.1080/02796015.2011.12087713

40.	McInnes, A., Humphries, T., Hogg-Johnson, S., & Tannock, R. (2003). *Listening Comprehension and Working Memory Are Impaired in Attention-Deficit Hyperactivity Disorder Irrespective of Language Impairment*.

41.	Miranda, A., Mercader, J., Fernández, M. I., & Colomer, C. (2017). Reading Performance of Young Adults with Adhd Diagnosed in Childhood: Relations with Executive Functioning. *Journal of Attention Disorders*, *21*(4), 294–304. https://doi.org/10.1177/1087054713507977

42.	Montes-Montes, R., Delgado-Lobete, L., & Rodríguez-Seoane, S. (2021). Developmental Coordination Disorder, Motor Performance, and Daily Participation in Children with Attention Deficit and Hyperactivity Disorder. *Children (Basel, Switzerland)*, *8*(3). cmedm (33804502). https://doi.org/10.3390/children8030187

43.	O’Brien, J. W., Dowell, L. R., Mostofsky, S. H., Denckla, M. B., & Mahone, E. M. (2010). Neuropsychological Profile of Executive Function in Girls with Attention-Deficit/Hyperactivity Disorder. *Archives of Clinical Neuropsychology*, *25*(7), 656–670. https://doi.org/10.1093/arclin/acq050

44.	Pagirsky, M. S., Koriakin, T. A., Avitia, M., Costa, M., Marchis, L., Maykel, C., Sassu, K., Bray, M. A., & Pan, X. (2017). Do the kinds of achievement errors made by students diagnosed with ADHD vary as a function of their reading ability? *Journal of Psychoeducational Assessment*, *35*(1–2), 124–137. psyh (2017-05805-009). https://doi.org/10.1177/0734282916669020

45.	Parke, E. M., Becker, M. L., Graves, S. J., Baily, A. R., Paul, M. G., Freeman, A. J., & Allen, D. N. (2021). Social Cognition in Children With ADHD. *Journal of Attention Disorders*, *25*(4), 519–529. https://doi.org/10.1177/1087054718816157

46.	Pineda, D., Ardila, A., Rosselli, M., Cadavid, C., Mancheno, S., & Mejia, S. (1998). Executive Dysfunctions in Children with Attention Deficit Hyperactivity Disorder. *International Journal of Neuroscience*, *96*(3–4), 177–196. https://doi.org/10.3109/00207459808986466

47.	Razjouan, K., Hosseinzadeh, M., Zahed, G., Khademi, M., Davari, R., & Arabgol, F. (2023). Theory of mind in adolescents with Attention-Deficit Hyperactivity Disorder: A cross-sectional study. *Iranian Journal of Child Neurology*, *17*(1), 81–90. cmedm (36721828). https://doi.org/10.22037/ijcn.v17i1.39094

48.	Rohlf, H., Jucksch, V., Gawrilow, C., Huss, M., Hein, J., Lehmkuhl, U., & Salbach-Andrae, H. (2012). Set shifting and working memory in adults with attention-deficit/hyperactivity disorder. *Journal of Neural Transmission*, *119*(1), 95–106. https://doi.org/10.1007/s00702-011-0660-3

49.	Rubia, K., Smith, A., & Taylor, E. (2007). Performance of Children with Attention Deficit Hyperactivity Disorder (ADHD) on a Test Battery of Impulsiveness. *Child Neuropsychology*, *13*(3), 276–304. https://doi.org/10.1080/09297040600770761

50.	Schachar, R., Tannock, R., Marriott, M., & Logan, G. (1995). Deficient inhibitory control in attention deficit hyperactivity disorder. *Journal of Abnormal Child Psychology*, *23*(4), 411–437. https://doi.org/10.1007/BF01447206

51.	Schulz, K. P., Fan, J., Tang, C. Y., Newcorn, J. H., Buchsbaum, M. S., Cheung, A. M., & Halperin, J. M. (2004). Response Inhibition in Adolescents Diagnosed with Attention Deficit Hyperactivity Disorder During Childhood: An Event-Related Fmri Study. *American Journal of Psychiatry*, *161*(9), 1650–1657. https://doi.org/10.1176/appi.ajp.161.9.1650

52.	Sciberras, E., Mueller, K. L., Efron, D., Bisset, M., Anderson, V., Jongeling, B., & Nicholson, J. M. (2014). *Language Problems in Children With ADHD: A Community-Based Study*. *133*(5).

53.	Seymour, K. E., Kim, K. L., Cushman, G. K., Puzia, M. E., Weissman, A. B., Galvan, T., & Dickstein, D. P. (2015). Affective processing bias in youth with primary bipolar disorder or primary attention-deficit/hyperactivity disorder. *European Child & Adolescent Psychiatry*, *24*(11), 1349–1359. psyh (2015-09523-001). https://doi.org/10.1007/s00787-015-0686-4

54.	Shallice, T., Marzocchi, G. M., Coser, S., Del Savio, M., Meuter, R. F., & Rumiati, R. I. (2002). Executive Function Profile of Children with Attention Deficit Hyperactivity Disorder. *Developmental Neuropsychology*, *21*(1), 43–71. https://doi.org/10.1207/S15326942DN2101_3

55.	Simone, A. N., Bédard, A.-C. V., Marks, D. J., & Halperin, J. M. (2016). Good Holders, Bad Shufflers: An Examination of Working Memory Processes and Modalities in Children with and without Attention-Deficit/Hyperactivity Disorder. *Journal of the International Neuropsychological Society : JINS*, *22*(1), 1–11. cmedm (26714882). https://doi.org/10.1017/S1355617715001010

56.	Singh, J., Arun, P., & Bajaj, M. K. (2021). Theory of Mind and Executive Functions in Children With Attention Deficit Hyperactivity Disorder and Specific Learning Disorder. *Indian Journal of Psychological Medicine*, *43*(5), 392–398. https://doi.org/10.1177/0253717621999807

57.	Sjöwall, D., & Thorell, L. B. (2014). Functional Impairments in Attention Deficit Hyperactivity Disorder: The Mediating Role of Neuropsychological Functioning. *Developmental Neuropsychology*, *39*(3), 187–204. https://doi.org/10.1080/87565641.2014.886691

58.	Skogli, E. W., Egeland, J., Andersen, P. N., Hovik, K. T., & Øie, M. (2014). Few Differences in Hot and Cold Executive Functions in Children and Adolescents with Combined and Inattentive Subtypes of Adhd. *Child Neuropsychology*, *20*(2), 162–181. https://doi.org/10.1080/09297049.2012.753998

59.	Song, Y. (2015). Cognitive Function in Attention Deficit Hyperactivity Disorder. In J. M. Norvilitis (Ed.), *ADHD - New Directions in Diagnosis and Treatment*. InTech. https://doi.org/10.5772/60785

60.	Soto, E. F., Irwin, L. N., Chan, E. S. M., Spiegel, J. A., & Kofler, M. J. (2021). Executive Functions and Writing Skills in Children with and Without ADHD. *Neuropsychology*, *35*(8), 792–808. https://doi.org/10.1037/neu0000769

61.	Stavro, G. M., Ettenhofer, M. L., & Nigg, J. T. (2007). Executive functions and adaptive functioning in young adult attention-deficit/hyperactivity disorder. *Journal of the International Neuropsychological Society*, *13*(02). https://doi.org/10.1017/S1355617707070348

62.	Thoma, P., Sonnenburg, S., Marcinkowski, N., Juckel, G., Edel, M.-A., & Suchan, B. (2020). Social problem solving in adult patients with attention deficit hyperactivity disorder. *Psychiatry Research*, *285*, 112721. https://doi.org/10.1016/j.psychres.2019.112721

63.	Toplak, M. E., Bucciarelli, S. M., Jain, U., & Tannock, R. (2008). Executive Functions: Performance-Based Measures and the Behavior Rating Inventory of Executive Function (BRIEF) in Adolescents with Attention Deficit/Hyperactivity Disorder (ADHD). *Child Neuropsychology*, *15*(1), 53–72. https://doi.org/10.1080/09297040802070929

64.	Uran, P., & Kılıç, B. G. (2015). Comparison of neuropsychological performances and behavioral patterns of children with attention deficit hyperactivity disorder and severe mood dysregulation. *European Child & Adolescent Psychiatry*, *24*(1), 21–30. https://doi.org/10.1007/s00787-014-0529-8

65.	Villa, M., Barriopedro Moro, M. I., & Ruiz Pérez, L. M. (2020). Motor competence difficulties and attention deficit and hyperactivity disorder (ADHD) among secondary students. *Cuadernos de Psicología Del Deporte*, *20*(2), 47–62. https://doi.org/10.6018/cpd.360491

66.	Weyandt, L., DuPaul, G. J., Verdi, G., Rossi, J. S., Swentosky, A. J., Vilardo, B. S., O’Dell, S. M., & Carson, K. S. (2013). The Performance of College Students with and without ADHD: Neuropsychological, Academic, and Psychosocial Functioning. *Journal of Psychopathology and Behavioral Assessment*, *35*(4), 421–435. https://doi.org/10.1007/s10862-013-9351-8

67.	Wodka, E. L., Mark Mahone, E., Blankner, J. G., Gidley Larson, J. C., Fotedar, S., Denckla, M. B., & Mostofsky, S. H. (2007). Evidence That Response Inhibition Is a Primary Deficit in ADHD. *Journal of Clinical and Experimental Neuropsychology*, *29*(4), 345–356. https://doi.org/10.1080/13803390600678046

68.	Wu, Z.-M., Wang, P., Liu, J., Liu, L., Cao, X.-L., Sun, L., Yang, L., Cao, Q.-J., Wang, Y.-F., & Yang, B.-R. (2023). The clinical, neuropsychological, and brain functional characteristics of the ADHD restrictive inattentive presentation. *Frontiers in Psychiatry*, *14*, 1099882. https://doi.org/10.3389/fpsyt.2023.1099882

69.	Xiao, T., Xiao, Z., Ke, X., Hong, S., Yang, H., Su, Y., Chu, K., Xiao, X., Shen, J., & Liu, Y. (2012). Response Inhibition Impairment in High Functioning Autism and Attention Deficit Hyperactivity Disorder: Evidence from Near-Infrared Spectroscopy Data. *PLoS ONE*, *7*(10), e46569. https://doi.org/10.1371/journal.pone.0046569

70.	Zhang, X., Wang, S., Liu, Q., Wu, C., Du, Y., Wang, Y., & Fang, J. (2023). Alerting network, cognitive flexibility in children with attention deficit hyperactivity disorder and the moderating effect of neuroticism. *Medicine*, *102*(41), e35583. https://doi.org/10.1097/MD.0000000000035583

71.	Agnihotri, S., Subramaniapillai, S., Keightley, M., Rasmussen, C., Cameron, D., Ryan, J., & Rovet, J. (2019). Everyday memory difficulties in children and adolescents with Fetal Alcohol Spectrum Disorder. *Developmental Neurorehabilitation*, *22*(7), 462–469. https://doi.org/10.1080/17518423.2018.1519608

72.	Burden, M. J., Andrew, C., Saint‐Amour, D., Meintjes, E. M., Molteno, C. D., Hoyme, H. E., Robinson, L. K., Khaole, N., Nelson, C. A., Jacobson, J. L., & Jacobson, S. W. (2009). The Effects of Fetal Alcohol Syndrome on Response Execution and Inhibition: An Event‐Related Potential Study. *Alcoholism: Clinical and Experimental Research*, *33*(11), 1994–2004. https://doi.org/10.1111/j.1530-0277.2009.01038.x

73.	Coles, C. D., Platzman, K. A., Lynch, M. E., & Freides, D. (2002). Auditory and Visual Sustained Attention in Adolescents Prenatally Exposed to Alcohol. *Alcoholism: Clinical and Experimental Research*, *26*(2), 263–271. https://doi.org/10.1111/j.1530-0277.2002.tb02533.x

74.	Crocker, N., Riley, E. P., & Mattson, S. N. (2015). Visual-spatial abilities relate to mathematics achievement in children with heavy prenatal alcohol exposure. *Neuropsychology*, *29*(1), 108–116. https://doi.org/10.1037/neu0000094

75.	Fagerlund, A., Autti-Rämö, I., Kalland, M., Santtila, P., Hoyme, E. H., Mattson, S. N., & Marit, M. (2012). Adaptive behaviour in children and adolescents with foetal alcohol spectrum disorders: A comparison with specific learning disability and typical development. *European Child & Adolescent Psychiatry*, *21*(4), 221–231. https://doi.org/10.1007/s00787-012-0256-y

76.	Furtado, E. F., & Roriz, S. T. D. S. (2016). Inattention and impulsivity associated with prenatal alcohol exposure in a prospective cohort study with 11-years-old Brazilian children. *European Child & Adolescent Psychiatry*, *25*(12), 1327–1335. https://doi.org/10.1007/s00787-016-0857-y

77.	Glass, L., Graham, D. M., Akshoomoff, N., & Mattson, S. N. (2015). Cognitive factors contributing to spelling performance in children with prenatal alcohol exposure. *Neuropsychology*, *29*(6), 817–828. https://doi.org/10.1037/neu0000185

78.	Green, C. R., Mihic, A. M., Nikkel, S. M., Stade, B. C., Rasmussen, C., Munoz, D. P., & Reynolds, J. N. (2009). Executive function deficits in children with fetal alcohol spectrum disorders (FASD) measured using the Cambridge Neuropsychological Tests Automated Battery (CANTAB). *Journal of Child Psychology and Psychiatry*, *50*(6), 688–697. https://doi.org/10.1111/j.1469-7610.2008.01990.x

79.	Gross, L. A., Moore, E. M., Wozniak, J. R., Coles, C. D., Kable, J. A., Sowell, E. R., Jones, K. L., Riley, E. P., & Mattson, S. N. (2018). Neural correlates of verbal memory in youth with heavy prenatal alcohol exposure. *Brain Imaging and Behavior*, *12*(3), 806–822. cmedm (28656347). https://doi.org/10.1007/s11682-017-9739-2

80.	Howell, K. K., Lynch, M. E., Platzman, K. A., Smith, G. H., & Coles, C. D. (2006). Prenatal Alcohol Exposure and Ability, Academic Achievement, and School Functioning in Adolescence: A Longitudinal Follow-Up. *Journal of Pediatric Psychology*, *31*(1), 116–126. https://doi.org/10.1093/jpepsy/jsj029

81.	Jirikowic, T., Olson, H. C., & Kartin, D. (2008). Sensory Processing, School Performance, and Adaptive Behavior of Young School-Age Children with Fetal Alcohol Spectrum Disorders. *Physical & Occupational Therapy In Pediatrics*, *28*(2), 117–136. https://doi.org/10.1080/01942630802031800

82.	Kaemingk, K. L., & Tanner Halverson, P. (2000). Spatial Memory Following Prenatal Alcohol Exposure: More Than a Material Specific Memory Deficit. *Child Neuropsychology*, *6*(2), 115–128. https://doi.org/10.1076/chin.6.2.115.7058

83.	Kerns, K. A., Siklos, S., Baker, L., & Müller, U. (2016). Emotion recognition in children with Fetal Alcohol Spectrum Disorders. *Child Neuropsychology*, *22*(3), 255–275. https://doi.org/10.1080/09297049.2014.993310

84.	Lindinger, N. M., Jacobson, S. W., Davidson, L., Conradie, S., Dodge, N. C., Molteno, C. D., Meintjes, E. M., Gaab, N., & Jacobson, J. L. (2022). Reading Impairment in Adolescents with Fetal Alcohol Spectrum Disorders. *Scientific Studies of Reading*, *26*(6), 469–488. https://doi.org/10.1080/10888438.2022.2054717

85.	Lindinger, N. M., Malcolm‐Smith, S., Dodge, N. C., Molteno, C. D., Thomas, K. G. F., Meintjes, E. M., Jacobson, J. L., & Jacobson, S. W. (2016). Theory of Mind in Children with Fetal Alcohol Spectrum Disorders. *Alcoholism: Clinical and Experimental Research*, *40*(2), 367–376. https://doi.org/10.1111/acer.12961

86.	Mattson, S. N., Calarco, K. E., & Lang, A. R. (2006). Focused and Shifting Attention in Children with Heavy Prenatal Alcohol Exposure. *Neuropsychology*, *20*(3), 361–369. https://doi.org/10.1037/0894-4105.20.3.361

87.	Mattson, S. N., & Roebuck, T. M. (2002). Acquisition and Retention of Verbal and Nonverbal Information in Children With Heavy Prenatal Alcohol Exposure. *Alcoholism: Clinical and Experimental Research*, *26*(6), 875–882. https://doi.org/10.1111/j.1530-0277.2002.tb02617.x

88.	Rasmussen, C., & Bisanz, J. (2011). The Relation Between Mathematics and Working Memory in Young Children With Fetal Alcohol Spectrum Disorders. *The Journal of Special Education*, *45*(3), 184–191. https://doi.org/10.1177/0022466909356110

89.	Rasmussen, C., Pei, J., Manji, S., Loomes, C., & Andrew, G. (2009). Memory Strategy Development in Children with Foetal Alcohol Spectrum Disorders. *Developmental Neurorehabilitation*, *12*(4), 207–214. https://doi.org/10.1080/17518420902980126

90.	Rasmussen, C., Wyper, K., & Talwar, V. (2009). The Relationship Between Theory of Mind and Executive Functions in Children with Fetal Alcohol Spectrum Disorders. *Journal of Clinical Pharmacology*, *16*(16).

91.	Thomas, S. E., Kelly, S. J., Mattson, S. N., & Riley, E. P. (1998). Comparison of Social Abilities of Children with Fetal Alcohol Syndrome to Those of Children with Similar IQ Scores and Normal Controls. *Alcoholism: Clinical and Experimental Research*, *22*(2), 528–533. https://doi.org/10.1111/j.1530-0277.1998.tb03684.x

92.	Vaurio, L., Riley, E. P., & Mattson, S. N. (2011). Neuropsychological Comparison of Children with Heavy Prenatal Alcohol Exposure and an IQ-Matched Comparison Group. *Journal of the International Neuropsychological Society*, *17*(3), 463–473. https://doi.org/10.1017/S1355617711000063

93.	Way, E. L., & Rojahn, J. (2012). Psycho-Social Characteristics of Children with Prenatal Alcohol Exposure, Compared to Children with Down Syndrome and Typical Children. *Journal of Developmental and Physical Disabilities*, *24*(3), 247–268. https://doi.org/10.1007/s10882-012-9269-1

94.	Wyper, K. R., & Rasmussen, C. R. (2011). Language Impairments in Children with Fetal Alcohol Spectrum Disorder. *Journal of Population Therapeutics and Clinical Pharmacology*, *18*(2), 364–376.

95.	Coles, C. D., Platzman, K. A., Raskind‐Hood, C. L., Brown, R. T., Falek, A., & Smith, I. E. (1997). A Comparison of Children Affected by Prenatal Alcohol Exposure and Attention Deficit, Hyperactivity Disorder. *Alcoholism: Clinical and Experimental Research*, *21*(1), 150–161. https://doi.org/10.1111/j.1530-0277.1997.tb03743.x

96.	Crocker, N., Vaurio, L., Riley, E. P., & Mattson, S. N. (2009). Comparison of Adaptive Behavior in Children With Heavy Prenatal Alcohol Exposure or Attention‐Deficit/Hyperactivity Disorder. *Alcoholism: Clinical and Experimental Research*, *33*(11), 2015–2023. https://doi.org/10.1111/j.1530-0277.2009.01040.x

97.	Crocker, N., Vaurio, L., Riley, E. P., & Mattson, S. N. (2011). Comparison of Verbal Learning and Memory in Children With Heavy Prenatal Alcohol Exposure or Attention‐Deficit/Hyperactivity Disorder. *Alcoholism: Clinical and Experimental Research*, *35*(6), 1114–1121. https://doi.org/10.1111/j.1530-0277.2011.01444.x

98.	Glass, L., Ware, A. L., Crocker, N., Deweese, B. N., Coles, C. D., Kable, J. A., May, P. A., Kalberg, W. O., Sowell, E. R., Jones, K. L., Riley, E. P., Mattson, S. N., & Collaborative Initiative on Fetal Alcohol Spectrum Disorders (CIFASD). (2013). Neuropsychological deficits associated with heavy prenatal alcohol exposure are not exacerbated by ADHD. *Neuropsychology*, *27*(6), 713–724. https://doi.org/10.1037/a0033994

99.	Greenbaum, R. L., Stevens, S. A., Nash, K., Koren, G., & Rovet, J. (2009). Social Cognitive and Emotion Processing Abilities of Children With Fetal Alcohol Spectrum Disorders: A Comparison With Attention Deficit Hyperactivity Disorder. *Alcoholism: Clinical and Experimental Research*, *33*(10), 1656–1670. https://doi.org/10.1111/j.1530-0277.2009.01003.x

100.	Kooistra, L., Ramage, B., Crawford, S., Cantell, M., Wormsbecker, S., Gibbard, B., & Kaplan, B. J. (2009). Can Attention Deficit Hyperactivity Disorder and Fetal Alcohol Spectrum Disorder be differentiated by motor and balance deficits? *Human Movement Science*, *28*(4), 529–542. https://doi.org/10.1016/j.humov.2009.01.007

101.	Kooistra, L., Crawford, S., Gibbard, B., Ramage, B., & Kaplan, B. J. (2010). Differentiating attention deficits in children with fetal alcohol spectrum disorder or attention-deficit-hyperactivity disorder: Attention Deficits in Fetal Alcohol Spectrum Disorder and ADHD. *Developmental Medicine & Child Neurology*, *52*(2), 205–211. https://doi.org/10.1111/j.1469-8749.2009.03352.x

102.	Kooistra, L., Crawford, S., Gibbard, B., Kaplan, B. J., & Fan, J. (2011). Comparing Attentional Networks in Fetal Alcohol Spectrum Disorder and the Inattentive and Combined Subtypes of Attention Deficit Hyperactivity Disorder. *Developmental Neuropsychology*, *36*(5), 566–577. https://doi.org/10.1080/87565641.2010.549978

103.	Vaurio, L., Riley, E. P., & Mattson, S. N. (2008). Differences in executive functioning in children with heavy prenatal alcohol exposure or attention-deficit/hyperactivity disorder. *Journal of the International Neuropsychological Society*, *14*(1), 119–129. https://doi.org/10.1017/S1355617708080144

104.	Ware, A. L., Crocker, N., O’Brien, J. W., Deweese, B. N., Roesch, S. C., Coles, C. D., Kable, J. A., May, P. A., Kalberg, W. O., Sowell, E. R., Jones, K. L., Riley, E. P., Mattson, S. N., & the CIFASD. (2012). Executive Function Predicts Adaptive Behavior in Children with Histories of Heavy Prenatal Alcohol Exposure and Attention‐Deficit/Hyperactivity Disorder. *Alcoholism: Clinical and Experimental Research*, *36*(8), 1431–1441. https://doi.org/10.1111/j.1530-0277.2011.01718.x
